# New insights into the coordination between the polymerization and 3′-5′ exonuclease activities in ϕ29 DNA polymerase

**DOI:** 10.1038/s41598-018-37513-7

**Published:** 2019-01-29

**Authors:** Alicia del Prado, Irene Rodríguez, José María Lázaro, María Moreno-Morcillo, Miguel de Vega, Margarita Salas

**Affiliations:** Centro de Biología Molecular “Severo Ochoa,” (Consejo Superior de Investigaciones Científicas-Universidad Autónoma de Madrid), Universidad Autónoma, Cantoblanco, 28049 Madrid, Spain

## Abstract

Bacteriophage ϕ29 DNA polymerase has two activities: DNA polymerization and 3′-5′ exonucleolysis governed by catalytic sites present in two structurally distant domains. These domains must work together to allow the correct replication of the template and to prevent the accumulation of errors in the newly synthesized DNA strand. ϕ29 DNA polymerase is endowed with a high processivity and strand displacement capacity together with a high fidelity. Previous studies of its crystallographic structure suggested possible interactions of residues of the exonuclease domain like the Gln180 with the fingers subdomain, or water mediated and direct hydrogen bond by the polar groups of residues Tyr101 and Thr189 that could stabilize DNA binding. To analyse their functional importance for the exonuclease activity of ϕ29 DNA polymerase we engineered mutations to encode amino acid substitutions. Our results confirm that both residues, Tyr101 and Thr189 are involved in the 3′-5′ exonuclease activity and in binding the dsDNA. In addition, Tyr101 is playing a role in processivity and Thr189 is an important determinant in the fidelity of the DNA polymerase. On the other hand, the biochemical characterization of the mutant derivatives of residue Gln180 showed how the mutations introduced enhanced the 3′-5′ exonuclease activity of the enzyme. A potential structural conformation prone to degrade the substrate is discussed.

## Introduction

Bacteriophage ϕ29 DNA polymerase belongs to the family B of DNA-dependent DNA polymerases^1^ and is fully responsible for the viral DNA replication^2^. It is endowed with specific properties that make it different from the rest of known replicases. First, ϕ29 DNA polymerase initiates DNA replication by using a terminal protein (TP) as primer^3^ bypassing the need for a primase. It has an intrinsic high processivity and strand displacement capacity, that allows it to replicate the entire genome from a single binding event without requiring the assistance of processivity or unwinding factors^4^. The strand displacement activity in ϕ29 DNA polymerase is more efficient than that of other replicative DNA polymerases like those of the bacteriophages T4 or T7^5^, allowing the ϕ29 polymerase to work as a hybrid polymerase helicase to couple efficiently DNA replication and unwinding activities within the same polypeptide^6^. Both properties, processivity and strand displacement allow a ϕ29 DNA polymerase molecule, after a transition stage during which a sequential switch from TP-priming to DNA-priming occurs, to replicate the entire viral genome from a single binding event^4^. Besides these features, ϕ29 DNA polymerase has a high fidelity due to high nucleotide insertion discrimination values (10^4^−10^6^) and to the 3′-5′ exonuclease activity that proofreads polymerization errors^7,8^.

ϕ29 DNA polymerase is the only member of the protein-primed subgroup of DNA polymerases whose structure has been solved. It has a N-terminal domain (residues 1–189) with the 3′-5′ exonuclease activity and a C-terminal domain (residues 190–572) with the polymerization activity^9^. The function of the 3′-5′ exonuclease activity of DNA polymerases is to remove nucleotides that have been incorrectly incorporated prior to their extension^10^, contributing around two orders of magnitude to its fidelity^11^. Although the polymerization and the exonuclease activities are governed by catalytic sites placed in two structurally distant domains^9^, both activities must act in concert to achieve a productive and accurate replication. When an incorrect nucleotide is incorporated, the 3′ terminus of the primer must be physically moved from the polymerase to the exonuclease active site for the removal of the misinserted nucleotide, and then the corrected primer returns to the polymerization active site^12^. Thus, the formation of an exonuclease complex requires the melting of the primer-end and its transfer to the exonuclease active site^13^. Previous results showed that ϕ29 DNA polymerase edits the polymerization errors using an intramolecular pathway, so the primer terminus moves from one active site to the other without dissociation from the DNA^14^.

**Figure 1:**
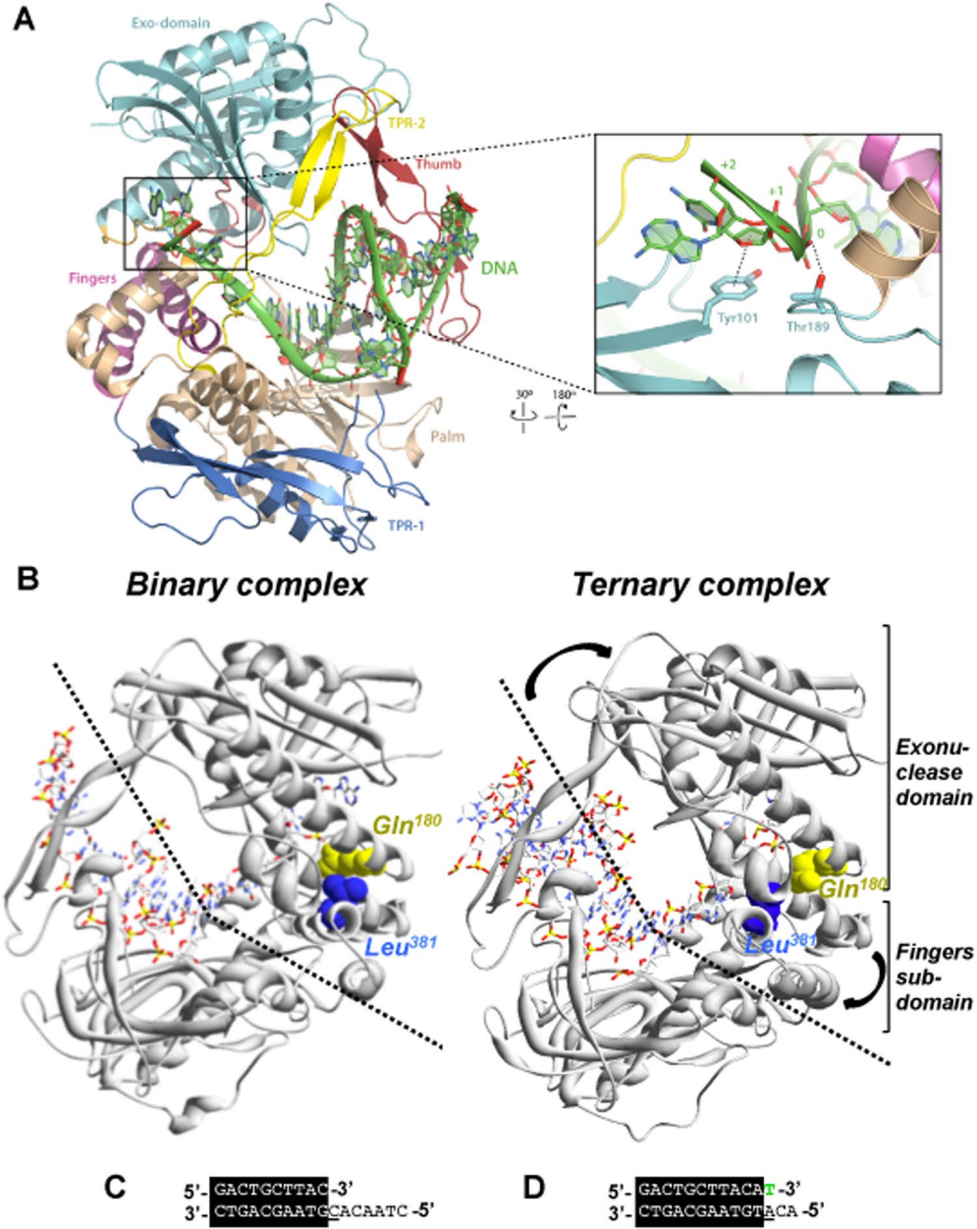
ϕ29 DNA polymerase structure. **(A)** Crystallographic data corresponding to ϕ29 DNA polymerase ternary complex are from Protein Data Bank (PDB) ID 2PYL^19^. The exonuclease domain is in cyan, the thumb in red, the palm in light brown, the fingers in magenta and the subdomains TPR1 and TPR2 in dark blue and yellow, respectively. Proposed interaction between the DNA and the Tyr101 and Thr189 residues (represented in sticks) **(B)** Changes in the position of the fingers subdomain and exonuclease domain in the binary (2PZS) and ternary (2PYL) complex. The Gln180 and Leu381 are depicted in yellow and blue spheres, respectively, to point out a possible interaction between both residues. **(C**,**D)** Primer/template molecules of the binary **(C)** and ternary **(D)** complexes. The 0 position on the template strand is underlined. The incoming dNTP of the ternary complex is indicated in green^19^. The (**A**) was made with Pymol software and (**B**) with Swiss PDB viewer software.

The polymerase domain can be subdivided in the universally conserved subdomains: palm, thumb and fingers by analogy to a semi-open right hand. The palm contains the catalytic and DNA ligand residues, the fingers the dNTP ligands and the thumb binds the DNA conferring stability to the primer^9^. There are also two insertions specifically present in the protein-primed DNA polymerases subgroup called Terminal Protein Regions 1 (TPR1) and 2 (TPR2). The TPR1 subdomain is involved in the interaction with the TP^15^. The palm, thumb and TPR2 subdomains form an internal ring-like structure that encircles the upstream duplex DNA at the polymerization active site^9^, providing the enzyme with its inherent high processivity^16^. The TPR2, palm and fingers subdomains, together with the exonuclease domain, form a tunnel that wraps the downstream template strand^9^. The narrow dimensions of this tunnel preclude the dsDNA binding, compelling the melting of the two strands to allow the template to reach the active site and providing to the polymerase the strand displacement capacity. Thus, the TPR2-exo tunnel is responsible for promoting the active destabilization of the duplex DNA^6^, in contrast with other polymerases whose strand displacement capacity relies on the fingers subdomain^17,18^ Residues in the downstream template tunnel interact with the two nucleotides that lie immediately downstream (+1 and +2) of the template nucleotide. Previous studies of the crystallographic structure of ϕ29 DNA polymerase suggested that water mediated and direct hydrogen bonds by the polar groups of Tyr101 and Thr189 were important for stabilizing DNA binding^19^ (see Fig. 1A).

A question that still remains open is how the polymerization and exonuclease domains coordinate to allow a mispaired 3′ terminus to reach the 3′-5′exonuclease active site in proofreading DNA polymerases. Inspection of the binary and ternary complexes of ϕ29 DNA polymerase shows how in the open conformation the fingers residue Leu381 is close to the exonuclease domain residue Gln180 (see Fig. 1B), allowing the concomitant movement of the exonuclease domain towards the polymerization one, a situation in which the exonuclease active site is catalytically competent. Such scenario could be modified once the fingers subdomain rotates into the closed catalytically competent polymerization conformation, promoting the exonuclease domain to adopt a more “opened” and maybe non-catalytically competent position respect to the polymerase domain. This last conformation could represent a premature exonucleolytic release of the incoming nucleotide.

The results presented in this paper by using ϕ29 DNA polymerase mutants show the importance of residues Tyr101 and Thr189, not only in the exonucleolytic activity and in DNA interaction, but also the involvement of Tyr101 in processivity and the role of Thr189 in the fidelity of the polymerization reaction. On the other hand, the change of the side chain of Gln180 could lead to an open position of the fingers that avoids the polymerization conformation and imposes an overdegradation of the primer. As mentioned before, after the incorporation of an incorrect nucleotide, the primer terminus has to move from the polymerization to the exonuclease active site to degrade only the misincorporated nucleotide and come back again to the polymerization active site to continue with the elongation of the primer. In this process, several interactions between the polymerase and the DNA must be broken and further reestablished to allow the polymerase to continue with the replication after the removal of a wrong nucleotide. In this work, the study of three residues of the exonuclease domain (Tyr101, Gln180 and Thr189) and their interactions with the DNA and with other residues of the polymerase allow us to improve the understanding of the molecular bases that govern the coordination between the exonuclease and polymerization activities.

## Results

### Site-directed mutagenesis of ϕ29 DNA polymerase

In order to ascertain the role of residues Tyr101 and Thr189 in the exonuclease activity through their interactions with the single stranded DNA (ssDNA) template near the primer end, as well as the potential involvement of residue Gln180 in the exonuclease activity, we engineered mutations to encode amino acid substitutions into alanine (Y101A, T189A and Q180A, in a non conservative change) and also into phenylalanine, serine and glutamic, respectively (Y101F, T189S and Q180E, in a more conservative change). The mutant derivatives were overexpressed and purified as described in Materials and Methods, and their catalytic efficiencies analysed by *in vitro* biochemical assays.

### 3′-5′ exonuclease activity and DNA binding capacity of ϕ29 DNA polymerase mutants

As mentioned before, ϕ29 DNA polymerase, as most DNA-dependent DNA polymerases, is endowed with two catalytic activities, DNA polymerization and 3′-5′ exonucleolysis. These activities are located in two structurally distant domains that must act in concert to achieve a productive and accurate replication reaction^9,20–23^. To study the equilibrium between both activities we used as substrate the hybrid primer/template (dsDNA) molecule sp1/sp1c + 6 (15/21 mer). This substrate contains a 6 nucleotides 5′-protuding end and therefore can be used as substrate for the exonuclease activity and for DNA-dependent polymerization. We can analyse the balance between both activities as a function of dNTPs concentration. In the absence of nucleotides, the only bands that could be detected correspond to primer degradation products by the 3′-5′ exonuclease activity. As the concentration of the dNTPs provided increases, the exonuclease activity is progressively competed by the 5′−3′ polymerization, with net dNMP incorporation onto the labelled primer, allowing us to define the dNTPs concentration needed to obtain an efficient elongation for each mutant derivative (Pol/Exo ratio).

**Figure 2:**
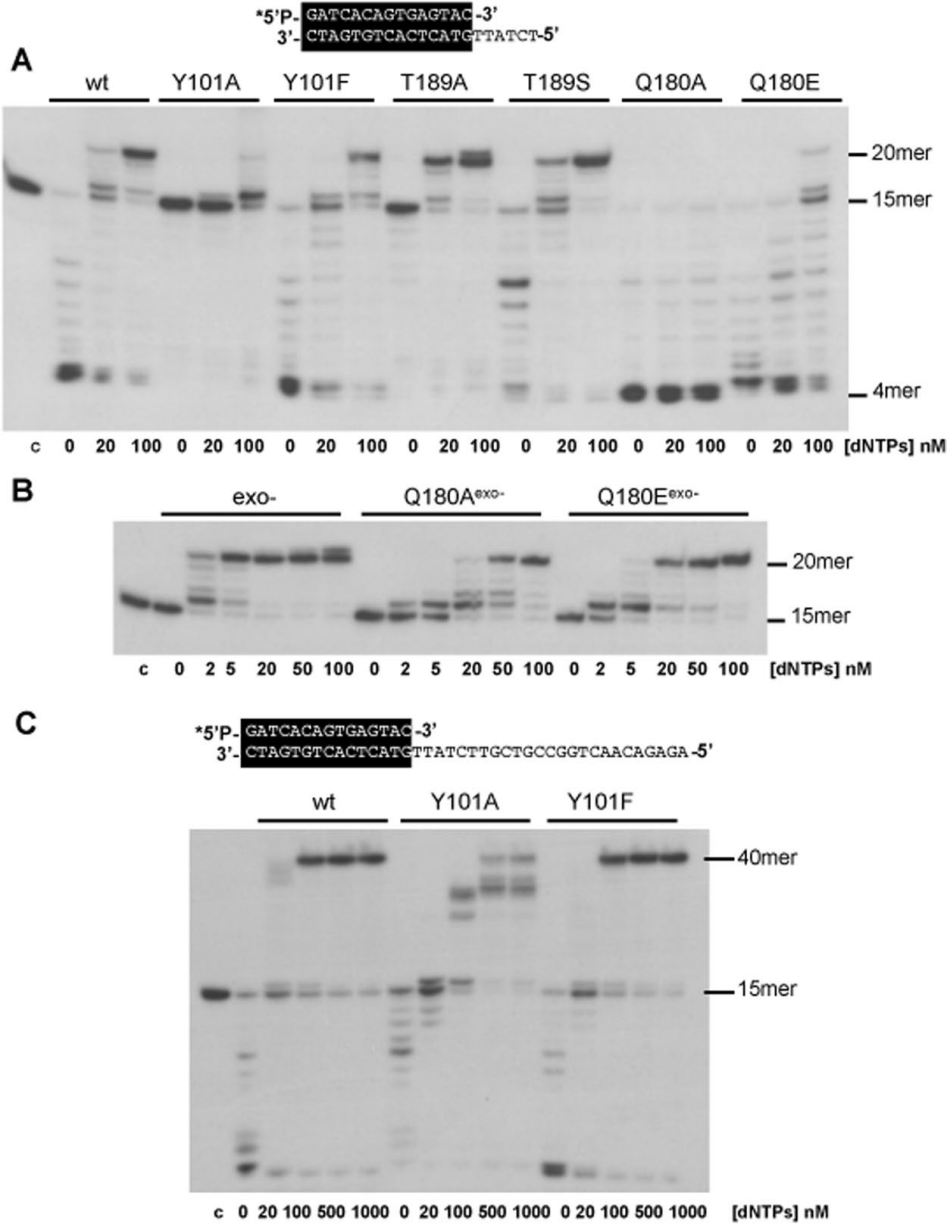
DNA polymerase/exonuclease coupled assay. (**A**) The assay was performed as described in Materials and Methods using the 5′-labelled primer/template molecule sp1/sp1c + 6 (15mer/21mer) depicted at the top of the figure, and the indicated concentration of dNTPs. Polymerization or 3-5′ exonucleolysis is detected as an increase or decrease, respectively, in the size (15mer) of the 5′-labelled primer. After incubation for five minutes at 25 °C, samples were analysed by 7 M urea-20% polyacrylamide gel electrophoresis and autoradiography. (**B**) DNA polymerization catalysed by wild-type exo- (D12A/D66A), Q180A^exo−^ (Q180A/D12A/D66A) and Q180E^exo−^ (Q180E/D12A/D66A) DNA polymerases. The assay was performed as described in (A), using as substrate the 5-labelled 15mer/21mer molecule and the indicated concentration of dNTPs. (**C**) DNA polymerase/exonuclease coupled assay with the wild-type DNA polymerase and the mutants Y101A and Y101F performed as described in (**A**) but using as substrate the 5′-labelled sp1/sp1c + 25 (15mer/40mer, depicted at the top of the figure) and the indicated amount of dNTPs. Asterisk indicates the 5′-^32^P-labelled end of the primer strand. c: control DNA.

As shown in Fig. 2A, mutant Y101F showed a wild-type exonuclease activity displaying a slightly reduced polymerization activity, as it requires 100 nM dNTPs to get a net elongation of the primer, in contrast to the wild-type enzyme that required 20–100 nM dNTPs. In the case of mutant Y101A, both the exonuclease and polymerization activities were strongly affected requiring a higher concentration of nucleotides (>100 nM) than the wild-type DNA polymerase to elongate the primer, suggesting that this mutation impairs both activities: exonucleolysis and polymerization. As it can be observed, mutant Y101A gives rise to a stop after the insertion of the first nucleotide. To ascertain whether such a blockage was due to an impaired translocation step, or to an early dissociation from the DNA, we carried out the assay both, in the presence of a higher dNTPs concentration (up to 1 μM) and a longer template strand. As shown in Fig. 2C, the mutant enzyme gave rise to elongation products longer than +1, however it stopped mainly four nucleotides before reaching the unit length. This result, together with the one shown in Fig. 2A suggest a reduced DNA binding capacity of the mutant once the length of the remaining template is shortened to four/five nucleotides. Mutants T189A and T189S showed a reduced exonuclease activity (especially mutant T189A), displacing the Pol/Exo ratio towards the polymerization (20 nM) (Fig. 2A). In the case of the mutants at residue Gln180, Q180A showed an enhanced exonuclease activity. Regarding the polymerization, whereas mutant Q180E required higher amount of nucleotides than the wild-type DNA polymerase to get elongation of the primer (100 nM), mutant Q180A was unable to render any polymerization product even at the highest amount of nucleotides assayed. To ascertain whether the absence of polymerization products by mutant Q180A was due to its strong exonuclease activity or to a polymerization defect we analysed the amount of dNTPs required to get a net elongation with the mutant deprived of exonuclease activity by including additional mutations at two conserved Asp residues (Asp12 and Asp66)^24^ of the exonuclease active site, obtaining the mutants Q180A^exo−^ and Q180E^exo−^. As shown in Fig. 2B, mutant Q180A^exo−^ (20–50 nM) and to a lower extent Q180E^exo−^ (5–20 nM) required a higher dNTPs concentration than the exo- (D12A/D66A mutant) DNA polymerase (2–5 nM) to reach the 20 mer position, indicating an impaired polymerization activity.

**Figure 3:**
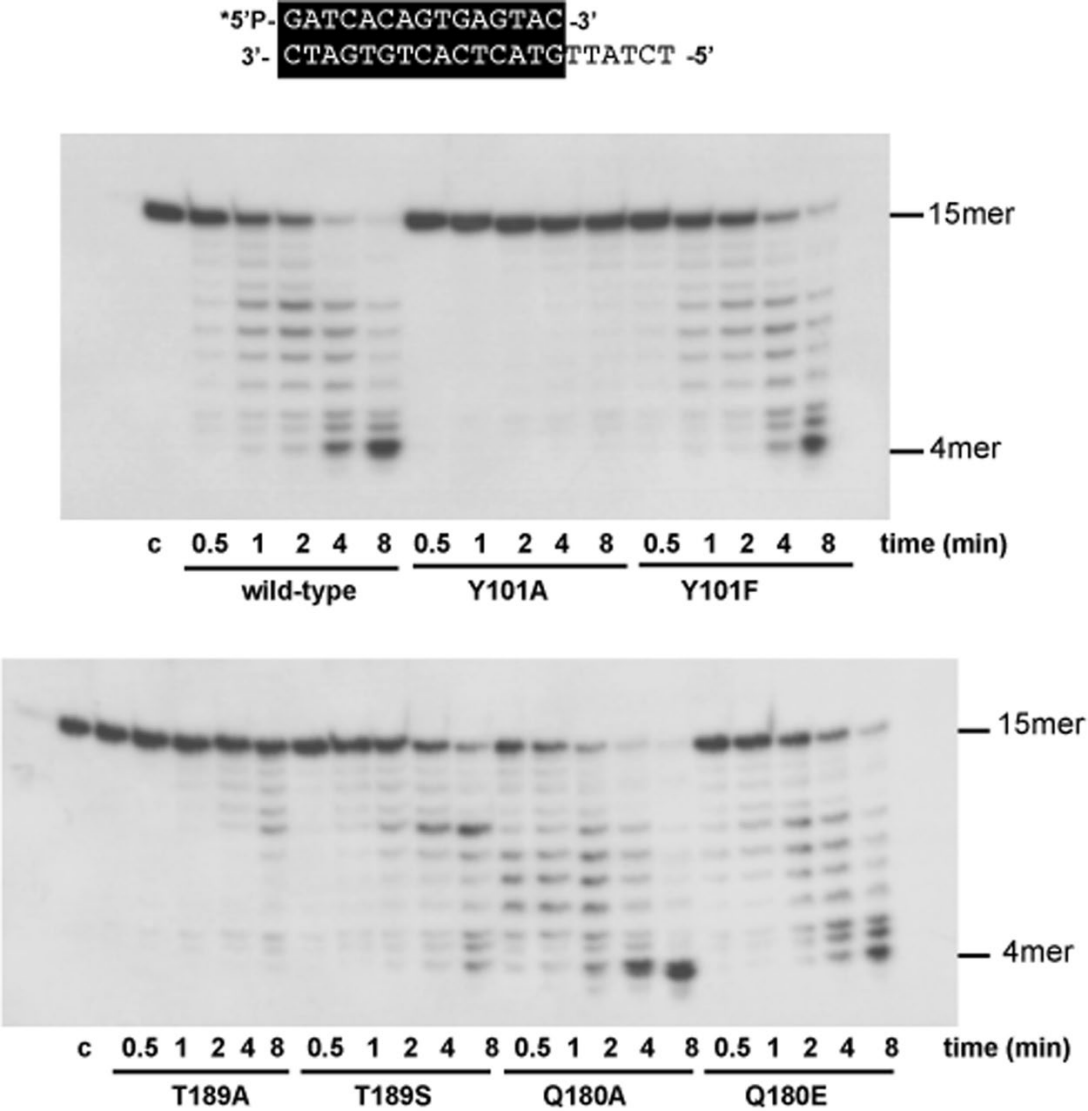
3′-5′-exonuclease activity of DNA polymerase mutants on dsDNA. The assay was carried out as described in Materials and Methods, using the 5′-labelled molecule sp1/sp1c + 6 (15mer/21mer) depicted at the top of the figure as dsDNA substrate. After incubation for the indicated times at 25 °C, degradation of the labelled DNA was analysed by electrophoresis in 7 M urea-20% polyacrylamide gels and autoradiography. The position of the 4mer degradation intermediate of the sp1 substrate (15mer) is indicated. Asterisk indicates the 5′-^32^P-labelled end of the primer strand. c: control DNA.

As most of the mutants showed an affected exonuclease activity (higher or lower than the wild-type DNA polymerase) using as substrate the primer/template structure, we analysed the degradation products in time course experiments (see Materials and Methods). As shown in Fig. 3 the most affected mutants were Y101A and T189A, in contrast to mutant Q180A whose exonuclease activity was enhanced respect to the wild-type one.

**Figure 4:**
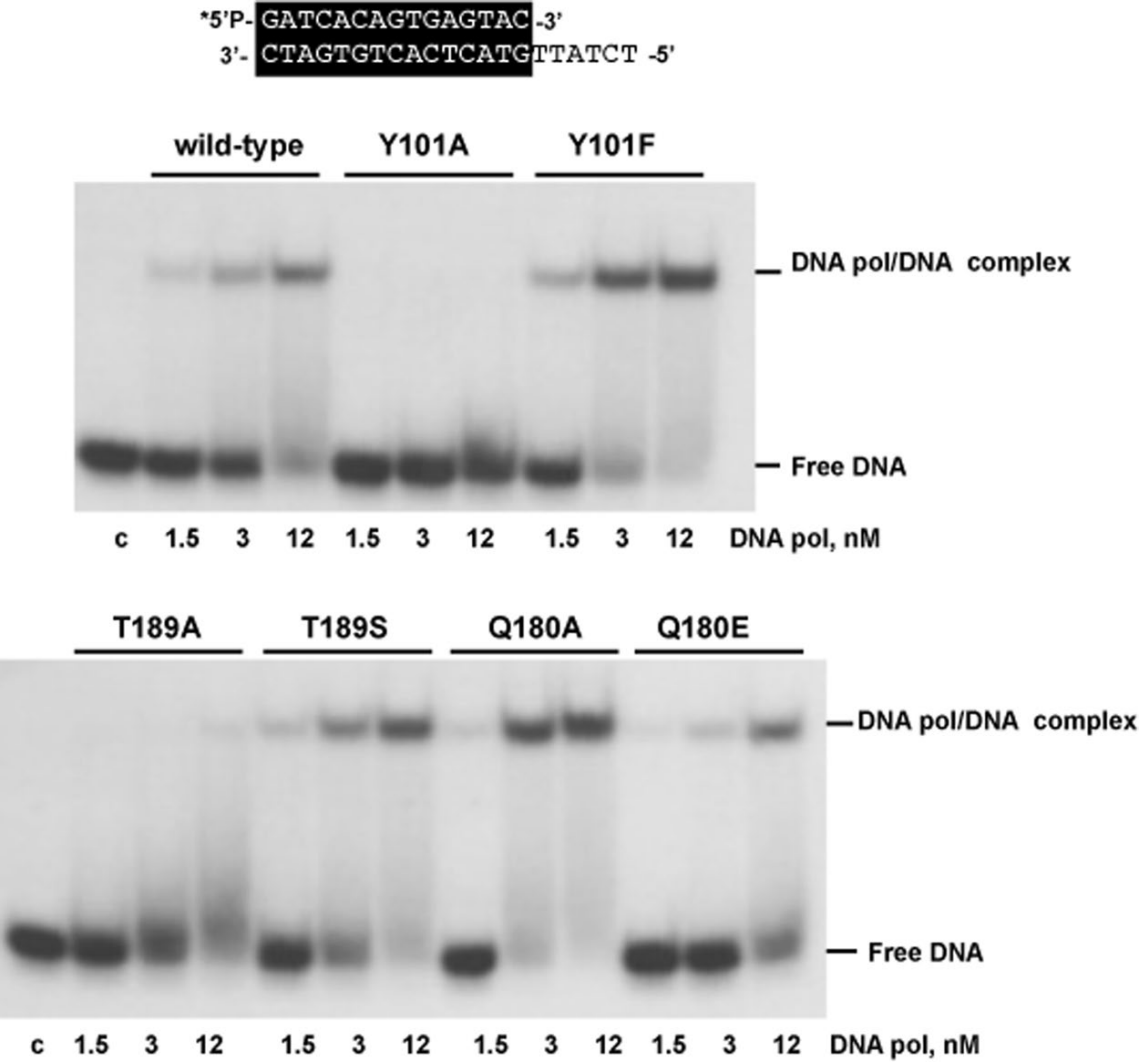
Gel retardation of template/primer molecules by ϕ29 DNA polymerase mutants. The 5′-labelled hybrid molecule sp1/sp1c + 6 (15mer/21mer) depicted at the top of the figure was incubated in the presence of the indicated amounts of either wild-type or mutant ϕ29 DNA polymerases, under the conditions described in Materials and Methods. After non-denaturing gel electrophoresis, the mobility of free DNA and the polymerase-DNA complex was detected by autoradiography. Asterisk indicates the 5′-^32^P-labelled end of the primer strand. c: control DNA.

To ascertain whether the low exonucleolytic levels displayed by mutants Y101A and T189A were due to an impaired DNA binding, gel retardation assays were carried out (see Materials and Methods). This assay was performed in the absence of divalent metal ions, to prevent the degradation of the dsDNA substrate. As shown in Fig. 4, the wild-type enzyme gave rise to a single retardation band, that has been interpreted as a stable complex competent for polymerization, in which the primer-terminus is stabilized at the polymerization active site^25^. As it can be observed, mutants Y101A and T189A did not bind stably the DNA substrate, explaining its affected exonuclease activity on a dsDNA substrate. The rest of the DNA polymerase mutants tested bound the dsDNA at a similar extent as the wild-type DNA polymerase with the only exception of mutant Q180A that showed a high dsDNA binding in accordance to its improved exonuclease activity. In the case of mutants Y101A and Y101F gel retardation assays were also performed with a longer protruding template (25 nt) (sp1/sp1c + 25) because the previous results suggested a reduced DNA binding capacity when the length of the template was short (6 nt). As shown in Supplementary Fig. 2 the mutant Y101A is moderately affected in DNA binding.

### The mutations introduced affect the polymerization activity

Regarding the exonuclease activity we can classify the mutants in two different groups according to their behaviour: impaired (mainly mutants Y101A and T189A) or enhanced (especially mutant Q180A) exonucleolysis. Focusing our attention in the first group, the affected exonuclease activity of mutant T189A unbalanced the equilibrium towards polymerization. But in the case of mutant Y101A, and in spite of having a reduced level of exonuclease activity, the balance between polymerization and exonuclease activities was not shifted towards the polymerization as could have been expected, indicating that this mutant was also impaired in the polymerization activity. With respect to mutant Q180A, that displayed a higher exonuclease activity than the wild-type DNA polymerase unbalancing the equilibrium towards the exonucleolysis, the results with the exonuclease deficient mutant showed that the polymerization activity was also impaired.

### ϕ29 DNA polymerase mutant T189A shows a diminished fidelity

**Figure 5:**
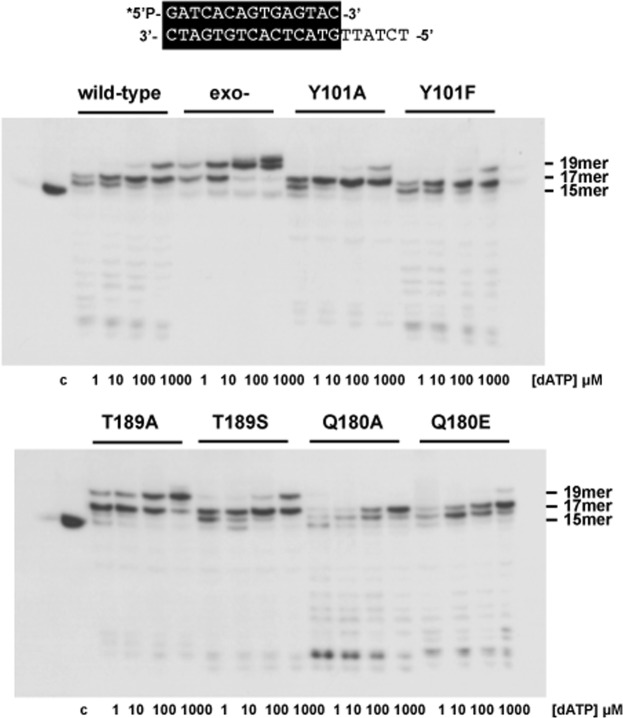
Misincorporation of ϕ29 DNA polymerase mutants. Conditions were essentially as described for the polymerization/exonuclease coupled assay on the primer/template molecule depicted at the top of the figure, but in this case, increasing concentrations of only dATP was added (see Materials and Methods). To prevent exonucleolytic degradation of the primer terminus, dCTP was added to 25 μM. After incubation at 25 °C for 5 min, samples were analysed by electrophoresis in 20% polyacrylamide gels containing 7 M urea. After autoradiography, misinsertion of dAMP at non-complementary positions was observed as the appearance of extension products of the 5′ labelled primer (15mer) larger than the correct 17mer extension product. The position corresponding to the unextended primer (15mer) and to extended products are indicated. Asterisk indicates the 5′-^32^P-labelled end of the primer strand. c: control DNA.

The reduced exonuclease activity exhibited by mutants Y101A and T189A could be compromising their editing function if they were able to produce a stable incorporation of incorrect nucleotides, affecting the fidelity of the polymerization. To address this issue, the capacity to promote stable incorporation of mismatched nucleotides during polymerization by the DNA polymerase mutants was studied by analysing the insertion of dAMP at the non-complementary positions 18 and 20, in comparison with the wild-type enzyme and the 3′-5′ exonuclease deficient mutant D12A/D66A^24^. Thus, we performed a misincorporation assay (see Materials and Methods), using as substrate the primer/template molecule sp1/sp1c +6 and increasing amounts of dATP as the sole deoxynucleotide. As shown in Fig. 5, under these conditions, the wild-type DNA polymerase was not able to produce a stable incorporation of dAMP at the non-complementary positions, the dAMP insertion occurring only opposite the two consecutives thymines at positions 16 and 17. Only in the presence of the highest dATP concentration (100 μM), the wild-type enzyme missinserted dAMP opposite position 18. As a control of the maximum misincorporation that could be reached in this assay, we used the exonuclease deficient mutant D12A/D66A. The presence of a band at position 19 indicated that dAMP misincorporation and further elongation of the mismatched primer-terminus had occurred even at the lowest dATP concentration (1 μM). In the case of mutants Y101A and Y101F the appearance of a band at position 19 only at the highest amount of nucleotide assayed, indicated that despite the decreased exonuclease activity, the fidelity was not affected, these mutants behaving like the wild-type enzyme. In these cases, to rule out that the wild-type fidelity showed by mutants Y101A and Y101F was not due to their defective replication when using short protruding templates (6 nt), we conducted a similar assay using a DNA substrate with a longer protruding template strand (25 nt) (Supplementary Fig. 4). As shown, the mutants did not display a reduced fidelity either under these conditions. Contrarily, as shown in Fig. 5, mutant T189A and to a lower extent mutant T189S, showed dAMP misincorporation at all the concentrations of dATP assayed, indicating a fidelity reduction relative to the wild-type DNA polymerase. In the case of the Gln mutants, especially Q180A, the strong exonuclease activity displaces the equilibrium towards exonucleolysis and precludes the study of the fidelity. Thus, incorporation of each of the four dNTPs was assayed with the exonuclease deficient mutants Q180A^exo−^ and Q180E^exo−^ using as substrate four template/primer structures depicted in Supplementary Fig. 3, covering the 16 possible template-substrate nucleotide pairs. As it can be observed, mutants Q180A^exo−^ and Q180E^exo−^ extended the primer strand mainly in the presence of the complementary (correct) nucleotide, performing DNA synthesis following the Watson-Crick base-pairing rules, the mutations not compromising the nucleotide insertion fidelity of the enzyme.

### ϕ29 DNA polymerase mutant Y101A shows a defective processivity

**Figure 6:**
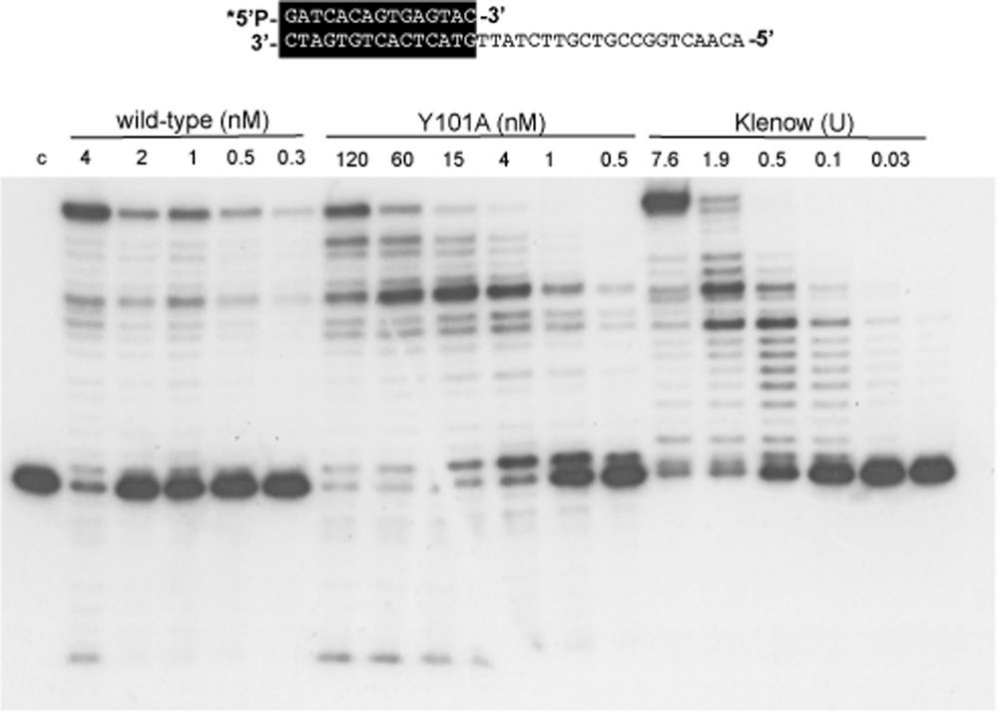
Processivity assay of ϕ29 DNA polymerase mutant Y101A. The assay was carried out as described in Materials and Methods by using a 5′ labelled sp1/sp1c + 18 (15/33 mer) depicted at the top of the figure as substrate, in the presence of the indicated concentrations of wild-type or mutant ϕ29 DNA polymerases. As a control of non-processive elongation, the Klenow DNA polymerase (units) was used. Asterisk indicates the 5′-^32^P-labelled end of the primer strand. c: control DNA.

As mentioned before, ϕ29 DNA polymerase is able to couple processive DNA synthesis to strand displacement. This enables the DNA polymerase to carry out the complete replication of the linear ϕ29 DNA in the absence of accessory proteins or helicases^4^. To ascertain whether the defective nucleotide insertion capacity opposite the latest nucleotides of the template exhibited by mutant Y101A could be due to a defective processivity of the enzyme, we analysed the chain length distributions during DNA polymerization as a function of the enzyme/DNA ratio (see Materials and Methods). As shown in Fig. 6, whereas the length of the elongation products made by the wild-type enzyme was not altered, the length of the products synthesized by mutant Y101A decreased with the enzyme/DNA ratio, showing a distributive polymerization pattern as it occurs with the Klenow polymerase used as a control. Regarding the rest of the mutants (see Supplementary Fig. 5), decreasing the enzyme/DNA ratio did not alter the length of the elongation products rendered by mutants T189A and T189S indicating a processive polymerization pattern. Conversely, and as expected, the length of the products synthesized by mutant Y101A and to a lower extent by Y101F decreased with the enzyme/DNA ratio, showing a distributive polymerization pattern. In the case of mutants Q180A and Q180E, the strong exonuclease activity compelled us to perform the assay using the exonuclease deficient variants (Q180A^exo−^ and Q180E^exo−^). Both mutants behaved as the exo- mutant D12A/D66A used as control, showing a processive polymerization pattern (Supplementary Fig. 5). The analysis of the processivity of mutant Y101A was also studied under single-turnover conditions in the presence of a 1000-fold excess of non-labelled substrate (challenger DNA). Under these conditions, all DNA polymerase molecules not bound to (or dissociated from) the 5′- labelled substrate molecules will be trapped by the challenger DNA, allowing the analysis of the polymerization from a single binding event. As shown in Supplementary Fig. 6 (challenger control panel) the amount of challenger DNA used was enough to trap all the DNA polymerase molecules since when it was simultaneously added to the reaction mixture no elongation of the labelled hybrid was observed. Although the wild-type ϕ29 DNA polymerase was not able to fully replicate the template in the presence of challenger DNA, mutant Y101A was only able to incorporate the first nucleotide when the challenger was added to the reaction. This would mean that mutant Y101A shows the minimal processivity that a DNA polymerase can exhibit (e.g. 1 nt).

### ϕ29 DNA polymerase mutants Q180A and Q180E exhibit an impaired strand displacement capacity

**Figure 7:**
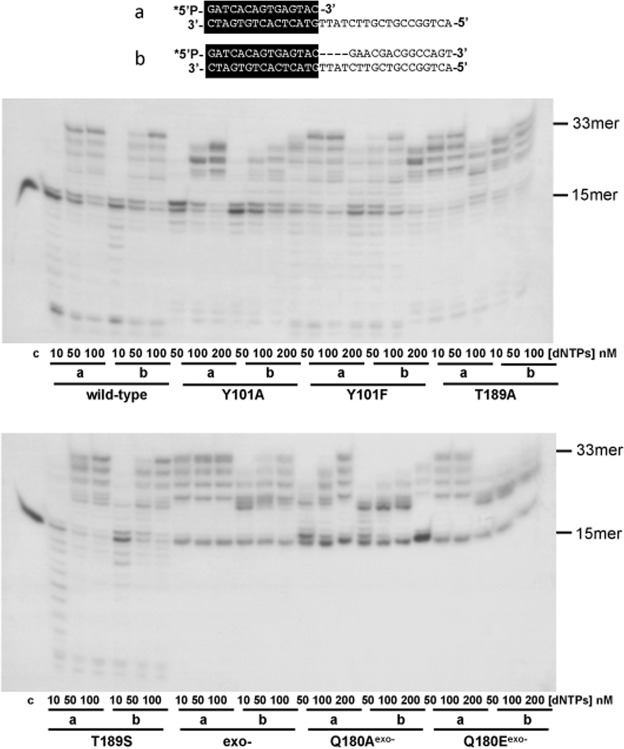
Assay of DNA polymerization coupled to strand displacement of ϕ29 DNA polymerase mutants. The assay was carried out on a 4 nucleotides gapped or non gapped substrate (depicted on the top of the figure) as described in Materials and Methods and the indicated amount of nucleotides. After incubation for 5 minutes at 25 °C, the reaction was stopped and samples were analysed by electrophoresis in 20% polyacrylamide gels containing 7 M urea and autoradiography. Asterisk indicates the 5′-^32^P-labelled end of the primer strand. c: control DNA.

To evaluate if the mutant derivatives are able to couple DNA synthesis with strand displacement, we analysed the extension of the primer on a gapped DNA molecule as described in Materials and Methods. In a parallel control experiment, we used the primer/template molecule sp1/sp1c + 18 (15/33 mer), which contains the same template strand as the gapped molecule but lacks a downstream non-template oligonucleotide (see Fig. 7). As expected, ϕ29 DNA polymerase was able to fully extend the primer (non-gapped substrate) and to fill the gap (4 nucleotides on gapped substrate), continuing DNA synthesis through the duplex region via strand displacement. The DNA polymerase mutants at residues Tyr101 and Thr189 were also able to fill the gap and to continue synthesis displacing the downstream oligonucleotide. To analyse the mutants Q180A^exo−^ and Q180E^exo−^, the mutant D12A/D66A was used as control. As shown in Fig. 7, although mutant D12A/D66A was able to produce strand displacement, required a higher dNTPs concentration than the wild-type enzyme in agreement with its reported defective strand displacement capacity^26^. Mutants Q180A^exo−^ and Q180E^exo−^ presented a stop after filling the gap displaying an even more defective strand displacement capacity than the D12A/D66A mutant.

## Discussion

Most DNA-dependent DNA polymerases have intrinsic 3′-5′ exonuclease activity to prevent nucleotide misincorporation errors during polymerization. This proofreading activity improves the fidelity of DNA replication (reviewed in^10,12^). The crystallographic analysis of the structure of several polymerases showed that the active sites for polymerization and 3′-5′ exonucleolysis are spatially distant. Despite the fact that both catalytic sites are separated they must act in concert to achieve a productive and accurate replication reaction^9,20,22,23,27–30^. The low catalytic constant exhibited by replicative DNA polymerases to elongate a mispaired 3′ terminus allows the melting of the two strands and further switching of the 3′ end from the polymerization to the exonuclease site. Once the 3′ nucleotide is released the primer end goes back to the pol site to resume polymerization (reviewed in^12^).

**Figure 8:**
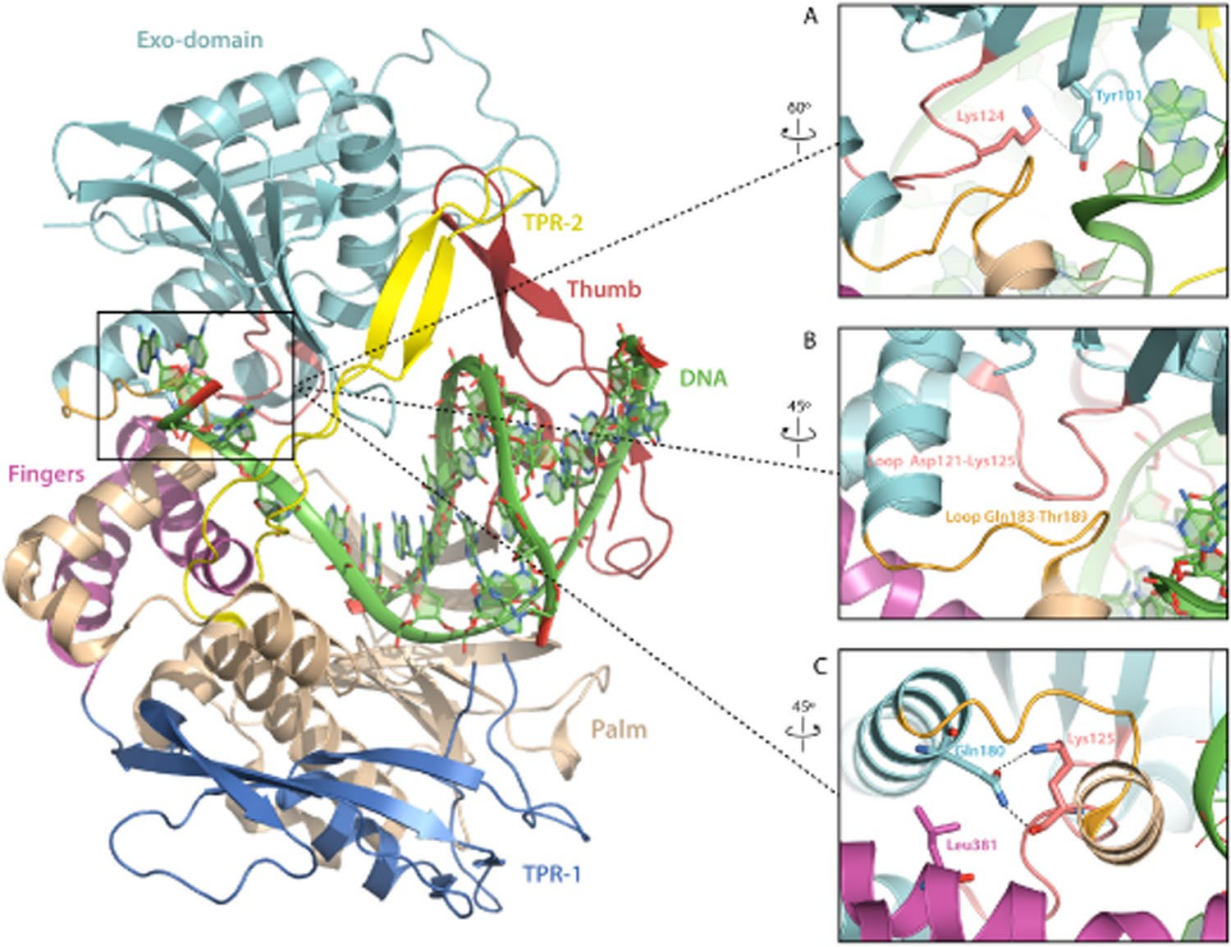
ϕ29 DNA polymerase structure. Crystallographic data corresponding to ϕ29 DNA polymerase binary complex are from Protein Data Bank (PDB) ID 2PZS^19^. The exonuclease domain is in cyan, the thumb in red, the palm in light brown, the fingers in magenta and the subdomains TPR1 and TPR2 in dark blue and yellow, respectively, like in Fig. 1. (**A**) Detail of the possible interaction between Y101 (coloured in blue) and Lys124 (coloured in light pink) **(B)** Detail of the interaction between the loop Gln183-Thr189 (coloured in orange) and the loop Asp121-Lys125 (coloured in light pink). **(C)** Detail of the potential interaction between the Gln180 (coloured in cyan) with the Leu381 (coloured in magenta) and the Lys125 (coloured in light pink) in the binary (**B**) complex. The Figure was made with Pymol software.

ϕ29 DNA polymerase is an outstanding enzyme that, unlike most replicases, is able to replicate the ϕ29 genome without unwinding proteins and processivity factors, due to its high processivity and strand displacement activity^4^. The resolution of its crystallographic structure allowed us to identify several hydrophilic residues of the exonuclease domain, as Tyr101 and Thr189, that have been predicted to interact with the single stranded template stabilizing the polar groups of the nucleotides by interacting with the backbone through water-mediated and direct hydrogen bonds. Additionally, Tyr101 stacks with the sugar of the +1 nucleotide of the template^19^. Our results show that mutants Y101A and T189A are unable to stabilise the DNA in the polymerization domain, and in the case of mutant Y101A, it showed a distributive pattern of polymerization in agreement with its role as template strand binding residue. This reduced processivity together with the affected stabilization of the template/primer at the polymerization active site impair the replication of the complete length of the template, possibly due to a prompt dissociation from the DNA when the size of the template is short, affecting the termination of the DNA replication. The nearly wild-type phenotype exhibited by mutant Y101F would be strengthening the importance of the stacking interaction between Tyr101 and the sugar moiety of the +1 nucleotide rather than the proposed polar interaction with the nucleotide backbone^19^. In addition, mutant Y101A exhibited a strongly reduced exonuclease activity. Such a defect could be explained by a disappearance of the stacking interaction with the DNA but also by the lack of the contact with Lys124 (see Fig. 8A). This lysine is interacting with the Asp121 that has been described as essential for the proper positioning of the primer end at the exo site^31^.

The polymerase mutants at residue Thr189 showed a reduced exonuclease activity compromising the editing function of the enzyme, mainly the T189A mutant that was able to produce a stable incorporation of incorrect nucleotides during polymerization, affecting the fidelity of the polymerization. The phenotype displayed by mutant T189S (conservative change) is similar to that of the wild-type enzyme, in contrast to the phenotype exhibited by mutant T189A (non conservative change). This could be evidencing the role of the hydroxyl group of the threonine in the interaction with the DNA. Furthermore, this threonine is located in the polymerase in a loop formed by residues from Gln183 to Thr189 that connects both protein domains, polymerase and exonuclease. This loop is making contacts with the loop formed by residues Asp121-Lys125^31^ (Fig. 8B); the mutation introduced could be destabilizing the interaction between both loops affecting the stabilisation of the primer end at the exonuclease active site by Asp121. Mutant T189A showed an unbalanced equilibrium towards the polymerization being residue Thr189 an important determinant in the fidelity of the DNA polymerase.

On the other hand, inspection of the ternary complexes of ϕ29 DNA polymerase shows how the open conformation of the fingers subdomain imposes a shift of the exonuclease domain towards the polymerase domain and that could be through a direct interaction between the fingers residue Leu381 and the exonuclease residue Gln180 (see Figs 1B and 8), a situation in which the exonuclease active site is catalytically competent. Such contacts are broken once the fingers subdomain rotates into the closed catalytically competent polymerization conformation, promoting the exonuclease domain to adopt a more “opened” and maybe non-catalytically competent position respect to the polymerase domain^19^. To test whether these contacts between the fingers subdomain and the exonuclease domain are playing a role in coordinating the synthetic and degradative activities of the polymerase we obtained mutants Q180A and Q180E. The biochemical characterization of these mutants showed how the mutations introduced enhanced the 3′-5′ exonuclease activity of the enzyme, not due to a polymerization defect but most probably to a structural conformation prone to degrade the substrate. To ascertain if the increased exonuclease activity is due to the lost of the interaction between Gln180 and Leu381 we engineered a new mutation to encode the amino acid substitution L381A. Unexpectedly, the Pol/Exo balance of mutant L381A was similar to that of the wild-type enzyme (Supplementary Fig. 7A), not showing an improved exonuclease activity (Supplementary Fig. 7B). This result could be indicating that in the movement of the exonuclease domain towards the polymerization one the interactions between Gln180 and Leu381 do not seem to play an essential role, and probably other interactions have a more important function. Therefore, the question is why mutant Q180A had an enhanced exonuclease activity. A detailed study of the structure of ϕ29 DNA polymerase allows to detect an interaction between Gln180 and Lys125 of the exonuclease domain (see Fig. 8C). The shortening of the side chain in mutant Q180A would avoid this interaction favouring the degradation of the DNA. On the contrary, the change into glutamic acid in mutant Q180E did not affect the exonuclease activity, presumably because the side chain could still interact with Lys125. The lack of the interaction with Lys125 could affect the stability of the loop formed by the residues from Asp121 to Lys125 forcing an overdegradation of the template^31^. In addition, the Gln180 variants also presented an affected strand displacement capacity as described in other residues of the ϕ29 DNA polymerase exonuclease domain^32–34^. As mentioned before, the exonuclease domain together with the TPR2 and palm subdomains, form a tunnel that would bind the downstream 5′ region of single-stranded template DNA^9,19^. The TPR2 subdomain acts as a wedge to promote strand displacement by separating the parental strands^16^. It has been shown how site directed mutants at the metal binding residues at the exonuclease active site also abolished the strand displacement capacity of the enzyme. These results, together with the defective strand displacement observed with mutants at residue Gln180 allow us to propose that the unwinding activity of the enzyme relies on a proper orientation of the TPR2 subdomain that depends of an intimate contact stablished with the exonuclease domain.

## Materials and Methods

### Nucleotides and DNAs

Unlabelled nucleotides were supplied by Amersham Pharmacia. [ϒ-^32^P]ATP (3000 Ci/mmol) was supplied by Perkin Elmer. Oligonucleotides were obtained from Invitrogen.

Oligonucleotide sp1 (15mer) (5′-GATCACAGTGAGTAC) was purified electrophoretically on 8 M urea-20% polyacrylamide gels and 5′-labelled with [γ-^32^P]ATP and phage T4 polynucleotide kinase. Labelled oligonucleotide 15mer was hybridized to oligonucleotide sp1c + 6 (21mer) (5′-TCTATTGTACTCACTGTGATC), sp1c + 18 (33mer) (5′-ACTGGCCGTCGTTCTATTGTACTCACTGTGATC), sp1c + 21 (5′-ACAACTGGCCGTCGTTCTATTGTACTCACTGTGATC) (36mer), sp1c + 25 (5′- AGAGACAACTGGCCGTCGTTCTATTGTACTCACTGTGATC) (40mer) or sp1c + 13 (28mer) with one of the four nucleotides (X) in the first templating base (5′ AGAAGTGTATCT**X**GTACTCACTGTGATC) in the presence of 0.2 M NaCl and 50 mM Tris-HCl, pH 7.5, resulting in primer/template structures. Oligonucleotide downstream (5′-GAACGACGGCCAGT), 5′-phosphorylated and complementary to the last 14 nucleotides of oligonucleotide sp1c + 18, was also hybridized to sp1/sp1c + 18 primer template to construct a 4-nt gapped structure with which to perform strand displacement assays.

### Proteins

DNA polymerase mutants were obtained using the QuikChange site-directed mutagenesis kit provided by Stratagene, using as template plasmid pJLPM (a derivative of pT7-4w2)^35^ containing the viral gene 2 that encodes the wild-type ϕ29 DNA polymerase, or plasmid pT7-3 that harbours ϕ29 DNA polymerase exonuclease deficient mutant D12A/D66A^24^ in the case of the mutants Q180A^exo−^ and Q180E^exo−^. The presence of the desired mutations, as well as the absence of additional ones was determined by sequencing the entire gene. DNA polymerase mutants were expressed in *Escherichia coli* BL21(DE3) cells and further purified essentially as described for the wild-type DNA polymerase^36^. The purified proteins are shown in Supplementary Fig. 1. The Klenow fragment of *E*.*coli* DNA polymerase was supplied by New England Biolabs.

### Polymerase/3′–5′ exonuclease (pol/exo) coupled assay

The primer/template molecule sp1/sp1c + 6 (15mer/21mer) contains a 6 nt 5′-protruding end (25 nt 5′protuding end when indicated) and therefore the primer strand can be used as substrate for DNA-dependent DNA polymerization and also for the exonuclease activity. The incubation mixture contained, in 12.5 µl, 50 mM Tris–HCl, pH 7.5, 10 mM MgCl_2_, 1 mM DTT, 4% (v/v) glycerol, 0.1 mg/ml BSA, 1.2 nM of 5′-labelled sp1/sp1c + 6 or sp1/sp1c + 25 substrate, 30 nM of wild-type or mutant ϕ29 DNA polymerases and the indicated increasing concentrations of the four dNTPs. After incubation for 5 min at 25 °C, the reaction was stopped by adding EDTA up to a final concentration of 10 mM. Samples were analysed by electrophoresis in 7 M urea-20% polyacrylamide gels and autoradiography. Polymerization or 3′-5′ exonucleolysis is detected as an increase or decrease, respectively, in the size (15mer) of the 5′-labelled primer.

### 3′-5′ Exonuclease assay

The incubation mixture contained, in 12.5 µl, 50 mM Tris-HCl, pH 7.5, 1 mM DTT, 4% (v/v) glycerol, 0.1 mg/ml BSA, 10 mM MgCl_2_, 5 nM of either the wild-type or the indicated ϕ29 DNA polymerase variants and 1.2 nM of 5′-labelled sp1/sp1c + 6 substrate. Samples were incubated at 25 **°**C for the indicated times and quenched by adding EDTA up to a final concentration of 10 mM. Reactions were analysed by electrophoresis in 7 M urea**-**20% polyacrylamide gels and autoradiography.

### DNA gel retardation assay

The incubation mixture contained, in a final volume of 20 µl, 12 mM Tris-HCl, pH 7.5, 1 mM EDTA, 20 mM (NH_4_)_2_SO_4_, 0.1 mg/ml BSA, 10 mM MgCl_2_, 0.7 nM of 5′ labelled sp1/sp1c + 6 (15/21 mer) or sp1/sp1c + 25 (15/40 mer) and the indicated amount of wild-type or mutant ϕ29 DNA polymerases. After incubation for 5 min at 4 °C, the samples were subjected to electrophoresis in precooled 4% (w/v) polyacrylamide gels (acrylamide/bis-acrylamide 80:1, w/w) containing 12 mM Tris-acetate, pH 7.5 and 1 mM EDTA, and run at 4 °C in the same buffer at 8 V/cm^37^. After autoradiography, ϕ29 DNA polymerase/DNA stable interaction was detected as a shift (retardation) in the migrating position of the labelled DNA.

### DNA replication misincorporation assay

Conditions were essentially as described above for the polymerization/exonuclease coupled assay on 5′ labelled spl/splc + 6 (15/21 mer) or sp1/sp1c + 25 (15/40 mer), but in this case increasing concentrations of only dATP, complementary to template positions 1, 2, 4 and 6, were added. To prevent exonucleolytic degradation of the primer-terminus, 25 μM dCTP was added. After incubation for 5 min at 30 °C, samples were analysed by 7 M urea-20% polyacrylamide gels. After autoradiography, misinsertion of dAMP at non-complementary positions is observed as the appearance of extension products of the 5′-labelled spl primer (15mer) longer than the correct 17mer extension product.

### Processivity assay

Processivity of polymerization was assessed by analysis of the length of replication products under decreasing DNA polymerase/DNA ratios. The 12.5 μl incubation mixture contained 50 mM Tris-HCl, pH 7.5, 10 mM MgCl_2_, 1 mM DTT, 4% (v/v) glycerol, 0.1 mg/ml BSA, 1.2 nM 5′-labelled 15/33 mer or 15/36 mer as indicated, 100 nM dNTPs, and the indicated decreasing amounts of either wild-type or mutant ϕ29 DNA polymerases or Klenow DNA polymerase. After incubation for 5 min at 25 °C, the reactions were stopped by adding EDTA up to a final concentration of 10 mM. Samples were analysed by 7 M urea- 20% polyacrylamide gels and autoradiography.

### Polymerization activity under single binding conditions

The incubation mixture contained in a final volume of 12.5 μl, 50 mM Tris-HCl, pH 7.5, 10 mM MgCl_2_, 1 mM DTT, 4% (v/v) glycerol, 0.1 mg/ml BSA, 1.2 nM 5′-labelled 15/36 mer, 100 nM dNTPs, and the indicated amounts of either wild-type or ϕ29 DNA polymerase mutant Y101A or Klenow DNA polymerase. The reaction was initiated by addition of the metal activator and a 1000-fold excess of non labelled substrate as challenger DNA. After incubation at 25 °C for 2.5 minutes the reactions were stopped by addition of EDTA to 10 mM. Samples were analysed by electrophoresis in 7 M urea-20% polyacrylamide gels and autoradiography.

### Strand displacement assay

A primer/template molecule with a gap of 4 nucleotides (see Nucleotides and DNAs) was used to study the strand displacement activity of ϕ29 DNA polymerase mutants. A primer/template construct (15/33 mer) that did not require strand displacement was also used as control. The 12.5 μl incubation mixture contained 50 mM Tris-HCl, pH 7.5, 10 mM MgCl_2_, 1 mM DTT, 4% (v/v) glycerol, 0.1 mg/ml BSA, 1.2 nM 5′-labelled 15/33 mer, 30 nM wild-type or mutant ϕ29 DNA polymerases, and the indicated concentration of dNTPs. After incubation for 5 min at 25 °C, the reaction was stopped by adding EDTA up to 10 mM. Samples were analysed by 7 M urea-20% polyacrylamide gels and autoradiography. The ability of the enzyme to carry out strand displacement was analysed by comparing the length of the elongation products when using the gapped and the nongapped primer/template molecules.

### Incorporation of the first nucleotide

The incubation mixture contained in 12.5 µl, 50 mM Tris-HCl, pH 7.5, 1 mM DTT, 4% (v/v) glycerol, 0.1 mg/ml BSA, 10 mM MgCl_2_ and 30 nM of ϕ29 DNA polymerase exonuclease deficient mutants D12A/D66A^24^, Q180A^exo−^ or Q180E^exo−^, 1.2 nM of the indicated 5′-labelled template/primer structure (differing in the first templating base) and 1 µM of the indicated dNTP. After incubation at 25 °C for 5 minutes, the reactions were stopped by the addition of EDTA up to 10 mM. Samples were analysed by 7 M urea, 20% polyacrylamide gels and autoradiography.

## Supplementary information

Supplementary Information
