# Supplementary material for: New insights into the coordination between the polymerization and 3′-5′ exonuclease activities in ϕ29 DNA polymerase
Source: Sci Rep. 2019 Jan 29;9:923. doi: 10.1038/s41598-018-37513-7 (PMC6351526; doi:10.1038/s41598-018-37513-7)
Supplement: Supplementary file 1 — Supplementary Information [file 41598_2018_37513_MOESM1_ESM.pdf]

Supplementary Information

"New insights into the coordination between the polymerization and 3'-5'exonuclease activities in ϕ29 DNA polymerase"

Alicia del Prado, Irene Rodríguez, José María Lázaro, María Moreno-Morcillo, Miguel de Vega and Margarita Salas

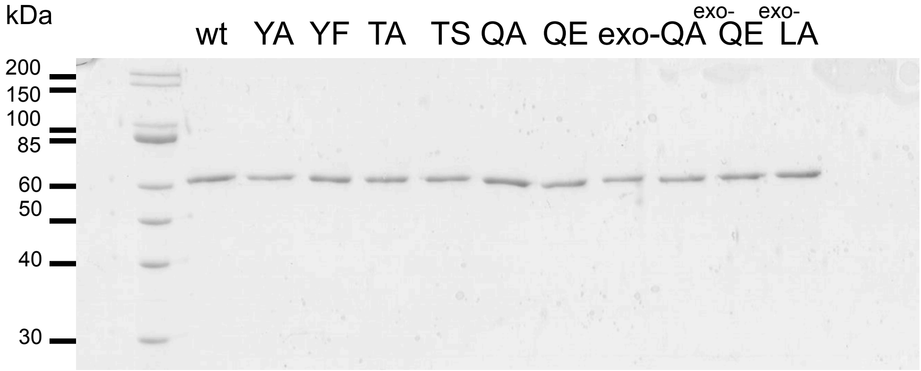

Supplementary Figure 1: Electrophoresis of purified ϕ29 DNA polymerase mutants. Aliquots (400 ng) of the purified preparations of wild-type polymerase and the indicated mutants were analysed in 12% SDS/PAGE. Polypeptides were visualized by staining the gel with Coomassie blue dye. The positions and size (in kDA) of the marker polypeptides are indicated on the left. wt (wild-type), YA (T101A), YF (Y101F), TA (T189A), TS (T189S), QA (Q180A), QE(Q180E), exo- (D12A/D66A), QA^{exo-} (Q180A/D12A/D66A), QE (Q180E/D12A/D66A), LA (L381A).

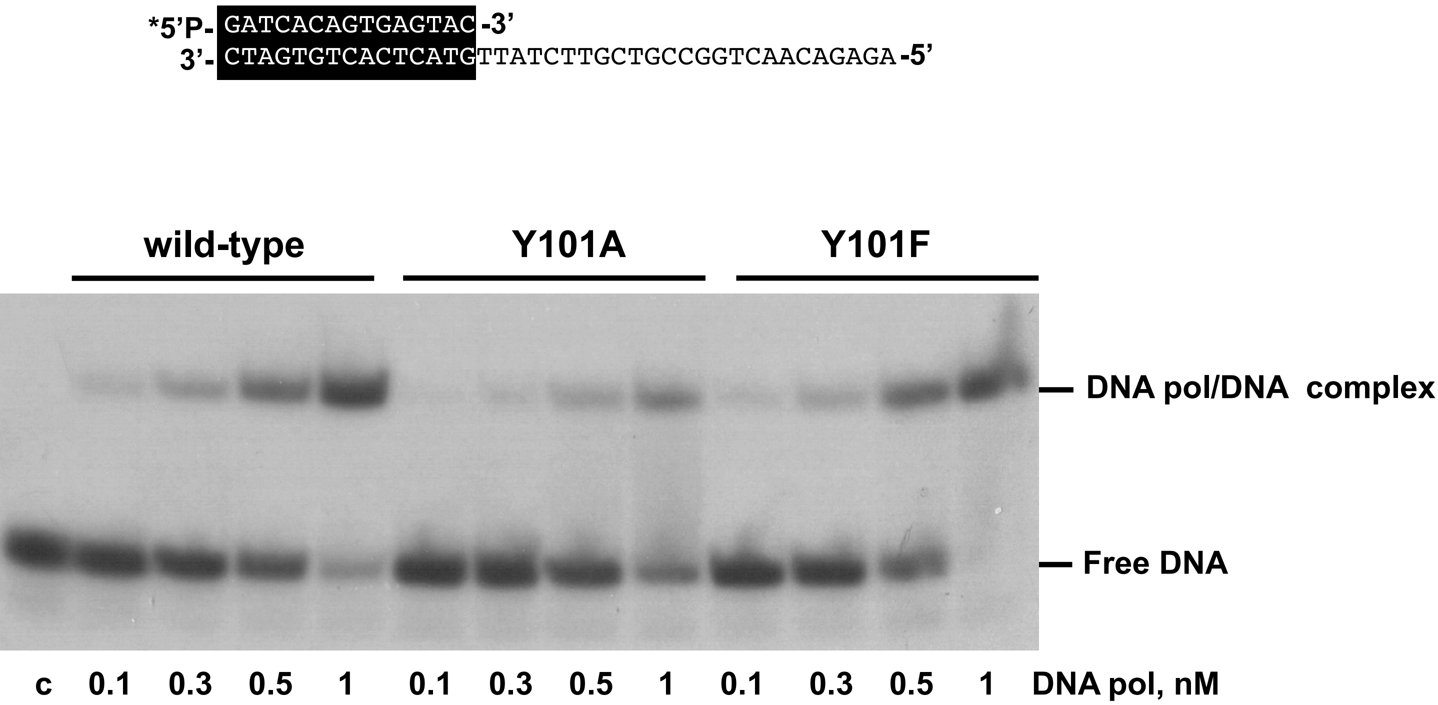

Supplementary Figure 2: Gel retardation of primer/template molecules by ϕ29 DNA polymerase mutants Y101A and Y101F. The 5′-labelled hybrid molecule sp1/sp1c+25 (15/40mer) depicted at the top of the figure was incubated in the presence of the indicated amounts of either wild-type or mutant ϕ29 DNA polymerases, under the conditions described in Materials and Methods. After non-denaturing gel electrophoresis, the mobility of free DNA and the polymerase-DNA complex was detected by autoradiography. Asterisk indicates the 5′ ^{32}P-labelled end of the primer strand. c: control DNA

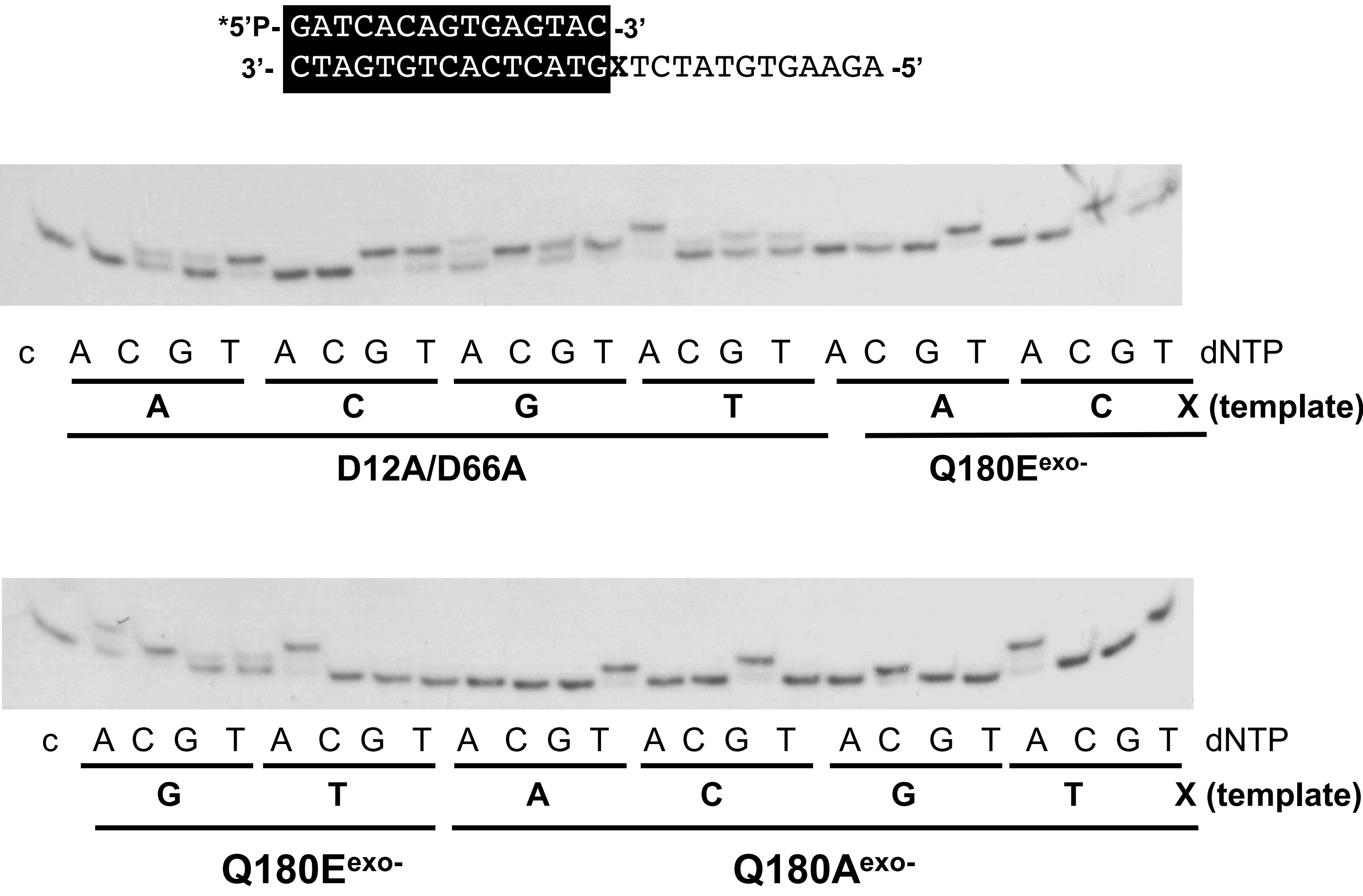

Supplementary Figure 3: Incorporation of the first nucleotide by ϕ29 DNA polymerase exonuclease deficient mutants D12A/D66A, Q180A^{exo-} and Q180E^{exo-}. Four different primer-template structures differing in the first templating base were used as substrate, as depicted at the top of the figure. After incubation for five minutes at 25 ºC with 1 μM of the indicated nucleotide, samples were analysed by 7 M urea-20% polyacrylamide gel electrophoresis and autoradiography. Asterisk indicates the 5’ ^{32}P-labelled end of the primer strand. c: control DNA

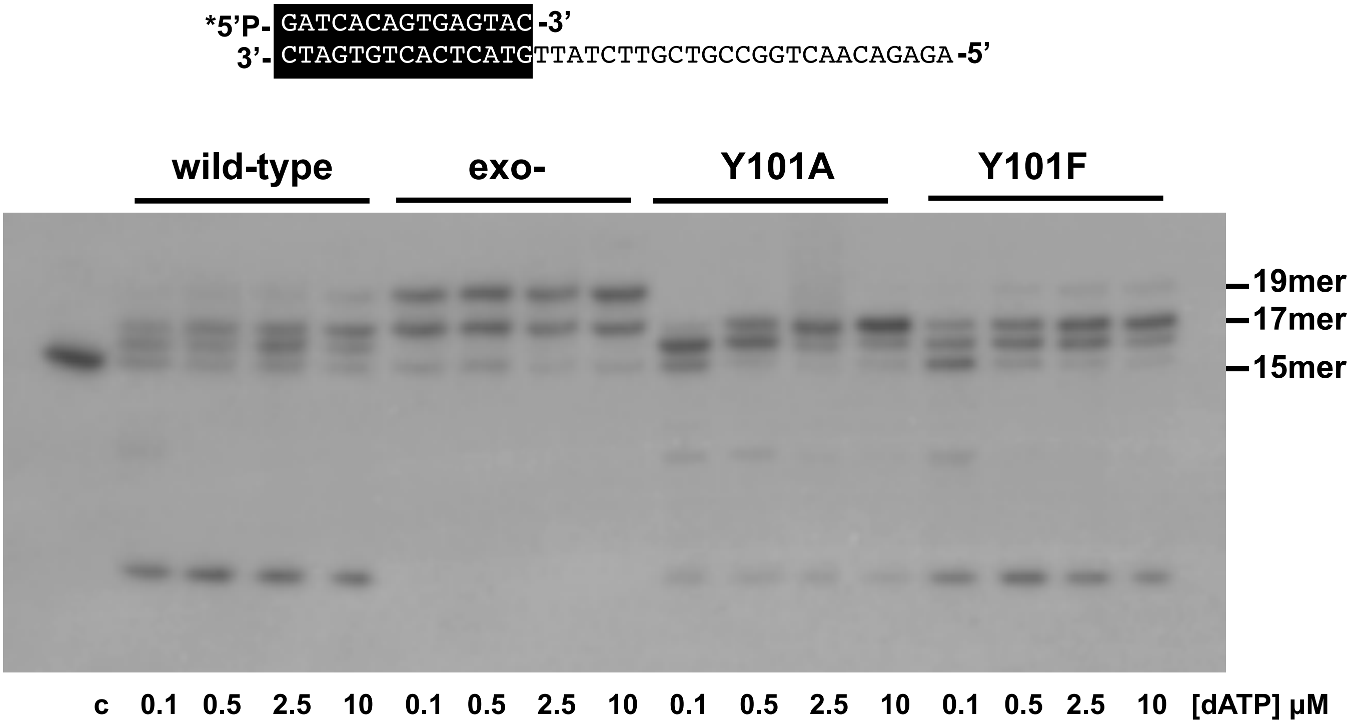

Supplementary Figure 4: Misincorporation of ϕ29 DNA polymerase mutants Y101A and Y101F. Conditions were essentially as described for the polymerization/exonuclease coupled assay on the primer/template molecule sp1/sp1c+25 (15/40 mer) depicted at the top of the figure, but using increasing concentrations of only dATP (see Materials and Methods). To prevent exonucleolytic degradation of the primer terminus, dCTP was added at 25 µM. After incubation at 25 °C for 5 min, samples were analysed by electrophoresis in 20% polyacrylamide gels containing 7 M urea. After autoradiography, misinsertion of dAMP at non-complementary positions was observed as the appearance of extension products of the 5´ labelled primer (15mer) larger than the correct 17mer extension product. The position corresponding to the unextended primer (15mer) and to extended products are indicated. Asterisk indicates the 5’ ^{32}P-labelled end of the primer strand. c: control DNA

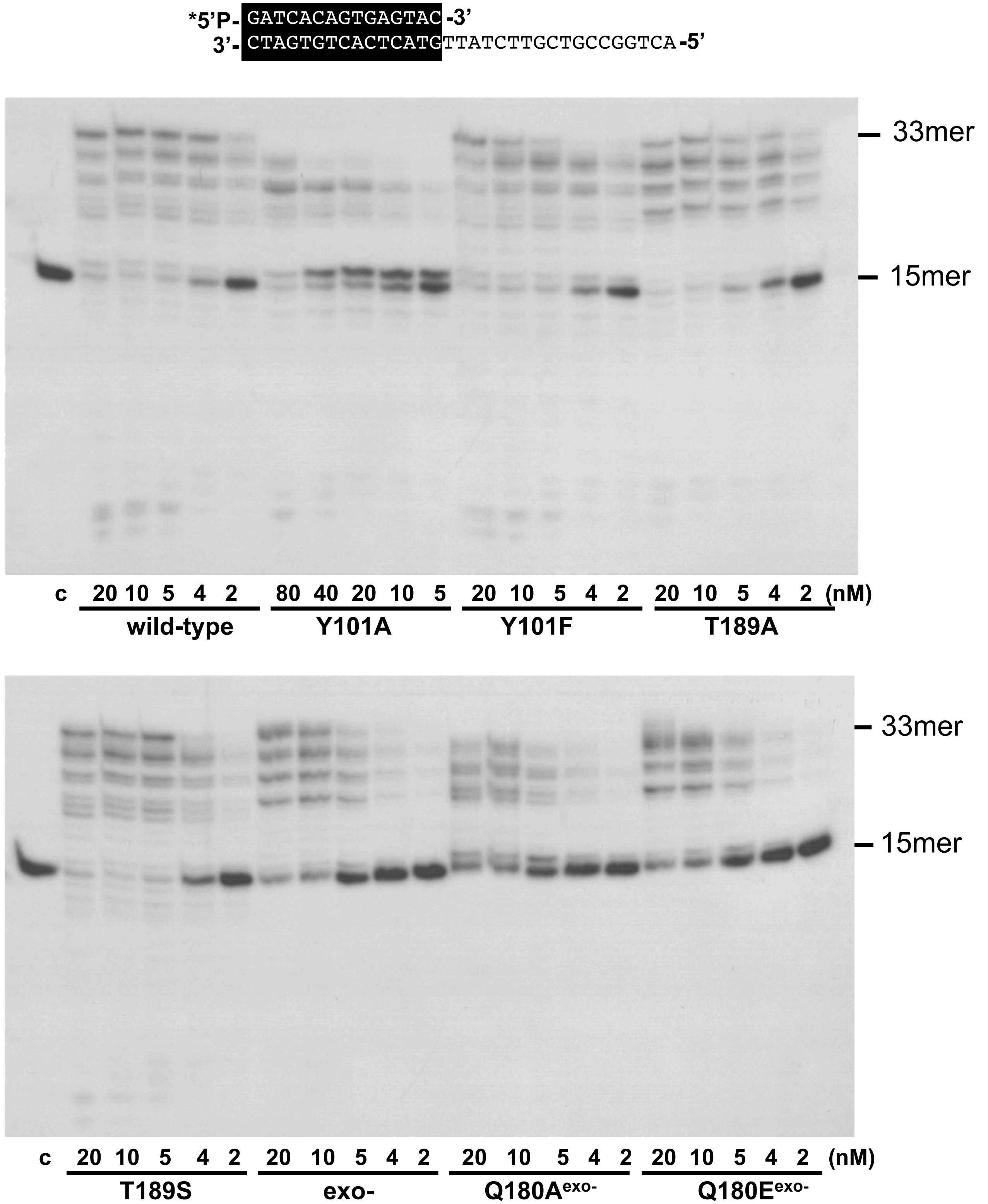

Supplementary Figure 5: Processivity assay of φ29 DNA polymerase mutants. The assay was carried out as described in Materials and Methods by using the 5´ labelled sp1/sp1c+18 (15/33 mer), depicted at the top of the figure as substrate, in the presence of the indicated concentrations of wild-type or mutant φ29 DNA polymerases. Asterisk indicates the 5'-^{32}P-labelled end of the primer strand. c: control DNA

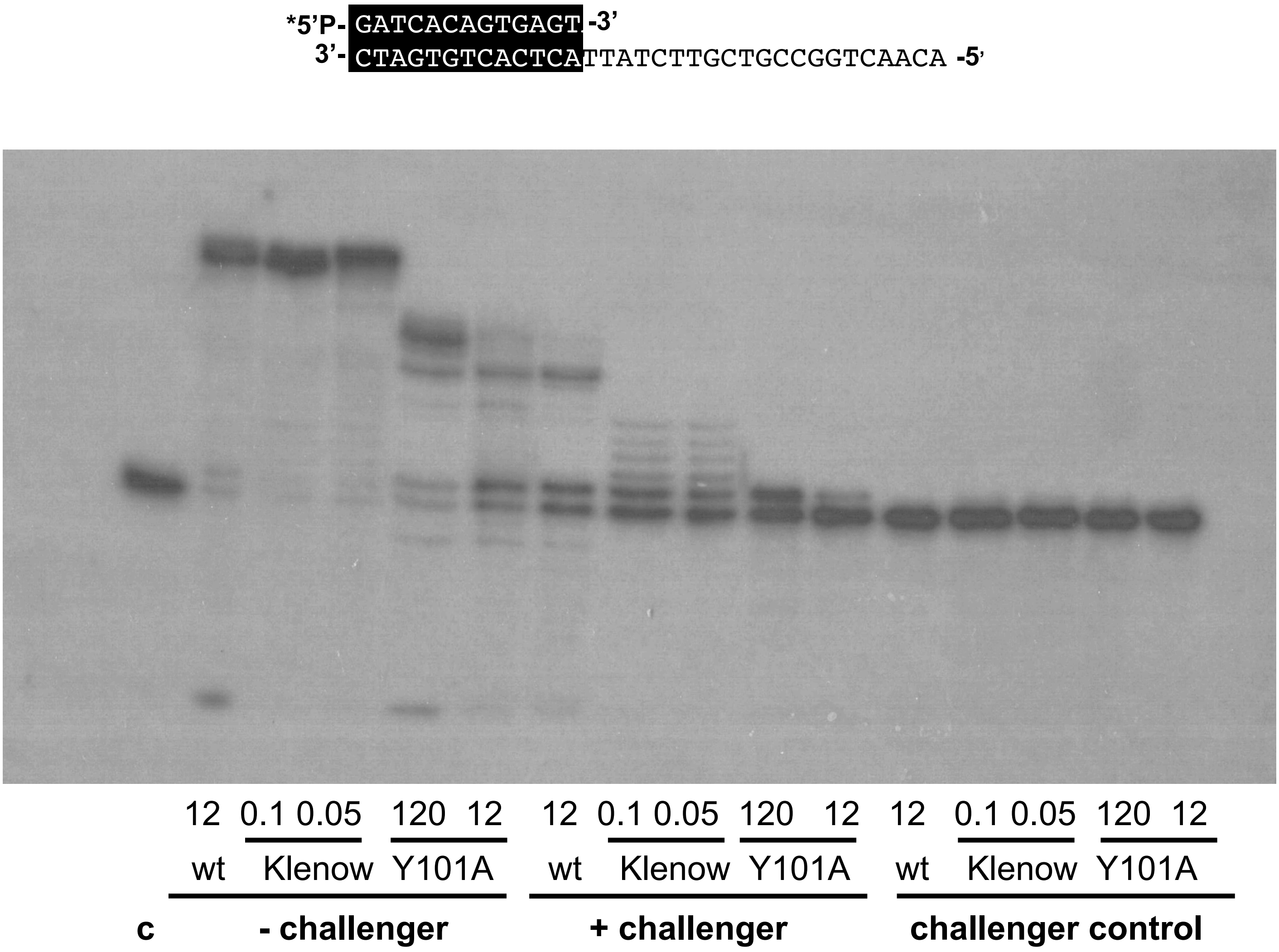

Supplementary Figure 6: Polymerization activity under single binding conditions. The assay was performed using as substrate the 5’ ^{32}P-labelled 15/36mer primer/template depicted on top of the figure and 100 nM dNTPs. The reaction was initiated by addition of the metal activator and a 1000-fold excess of non labelled substrate as challenger DNA (middle panel +challenger). After incubation at 25 ºC for 2.5 minutes the reactions were stopped by addition of EDTA to 10 mM and analysed by electrophoresis in 7 M urea-20% polyacrylamide gels and autoradiography. In the right panel (challenger DNA) the DNA polymerase was preincubated with a 1000-fold excess of the nonlabelled substrate as challenger DNA. In the left panel (-challenger) the experiment was performed without non labelled substrate. Asterisk indicates the 5’ ^{32}P-labelled end of the primer strand. The amount of protein is expressed in Units for Klenow and in nanoMolar for ϕ29 DNA polymerase and mutant Y101A. c: control DNA

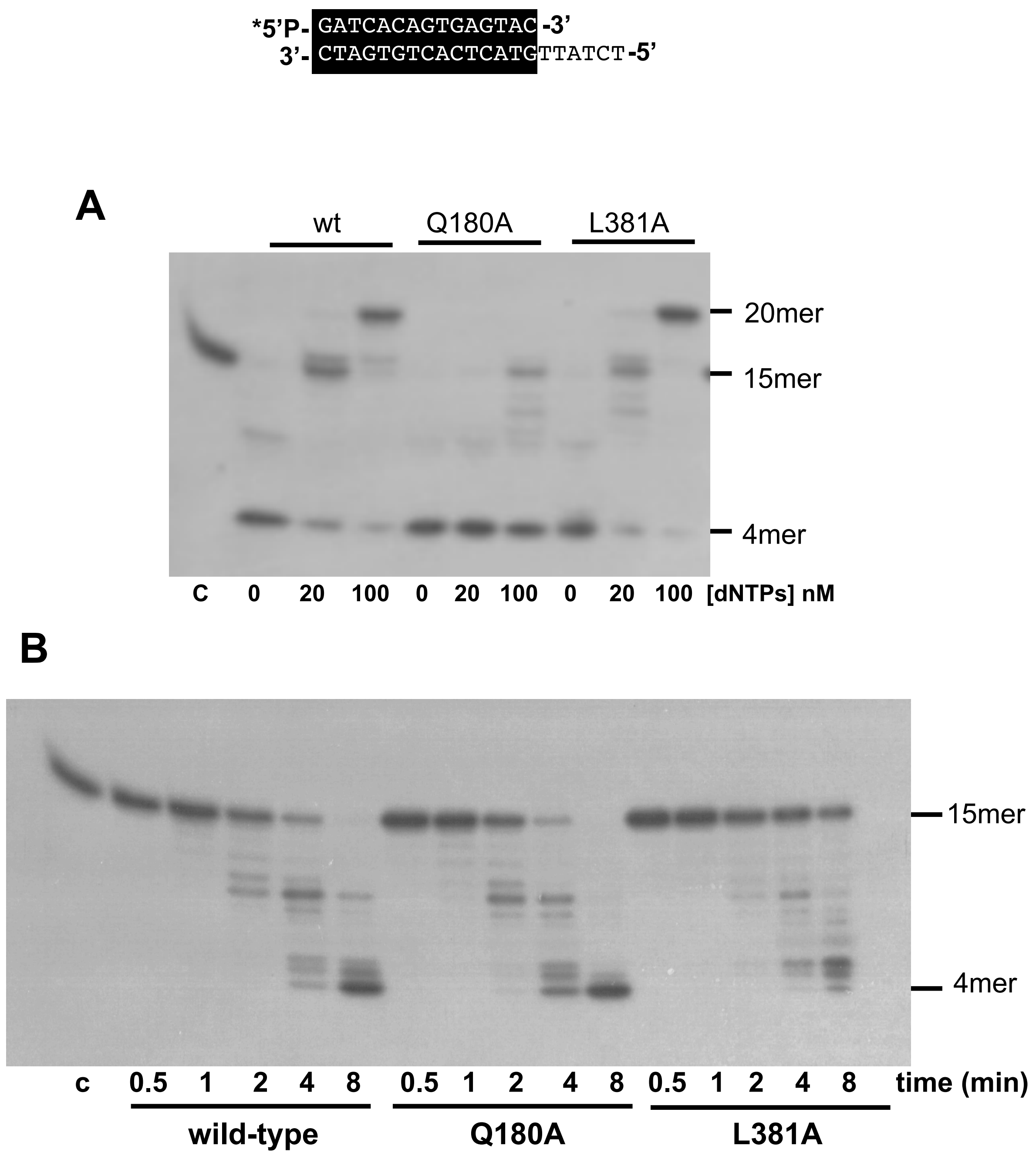

Supplementary Figure 7: DNA polymerase/exonuclease coupled assay. (A) 3´-5´-exonuclease activity of ϕ29 DNA polymerase mutants on dsDNA. The assay was performed as described in Materials and Methods using the 5'-labelled primer/template molecule sp1/sp1c+6 (15/21 mer) depicted at the top of the figure, and the indicated concentration of dNTPs (A) or without dNTPs (B). Polymerization or 3'-5' exonucleolysis is detected as an increase or decrease, respectively, in the size (15mer) of the 5´-labelled primer. After incubation for five minutes (A) or the indicated time (B) at 25 ºC, samples were analysed by 7 M urea-20% polyacrylamide gel electrophoresis and autoradiography. c: control DNA

Uncropped Figure 2A

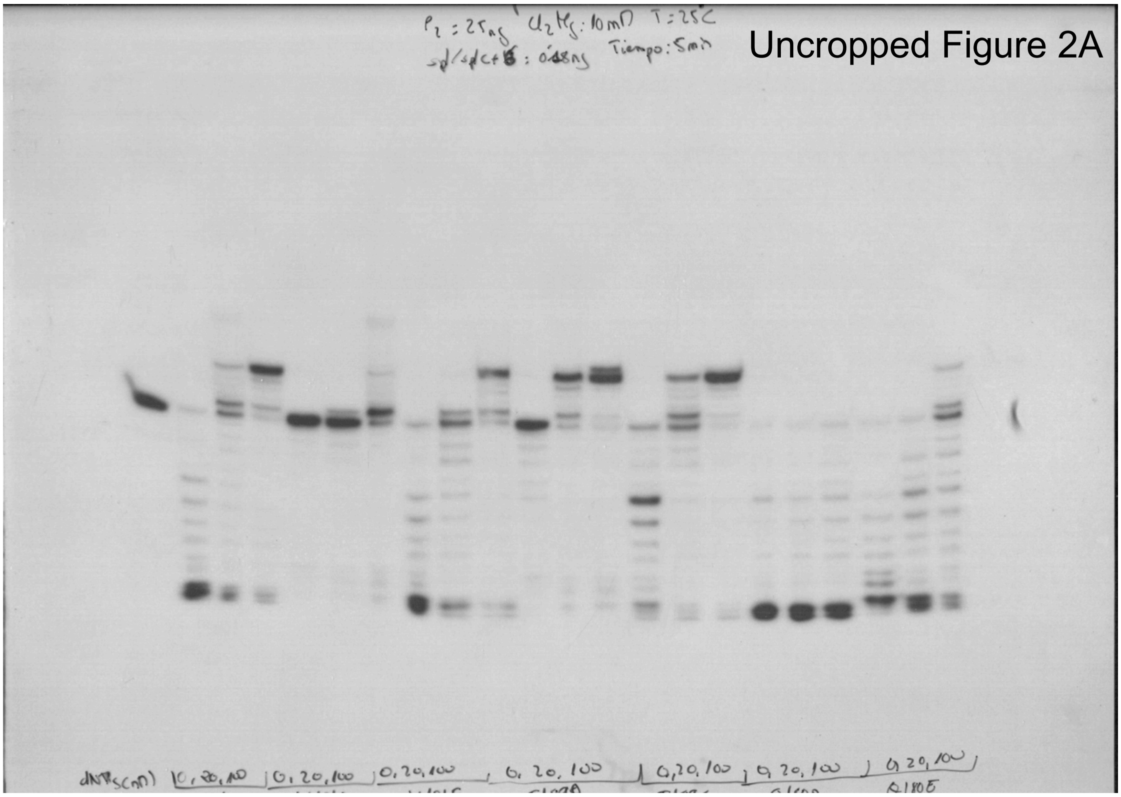

Uncropped Figure 2B

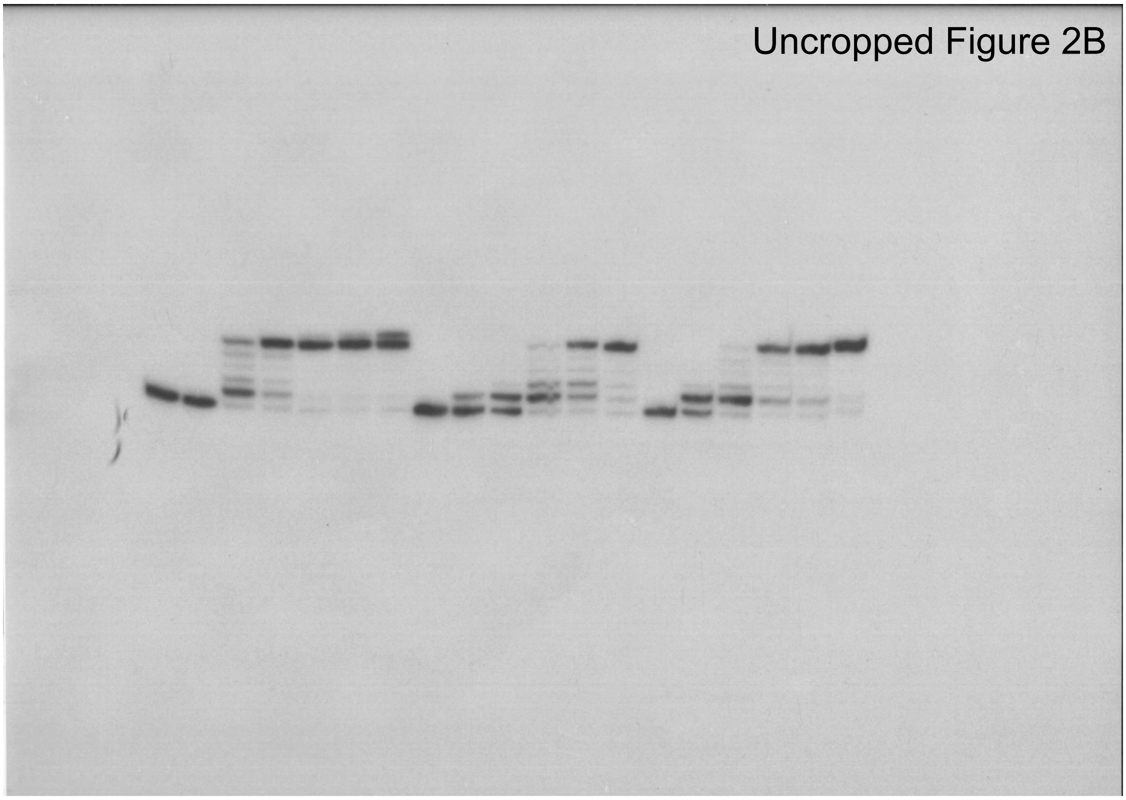

Supplementary Figure 8: Uncropped gels for Figure 2

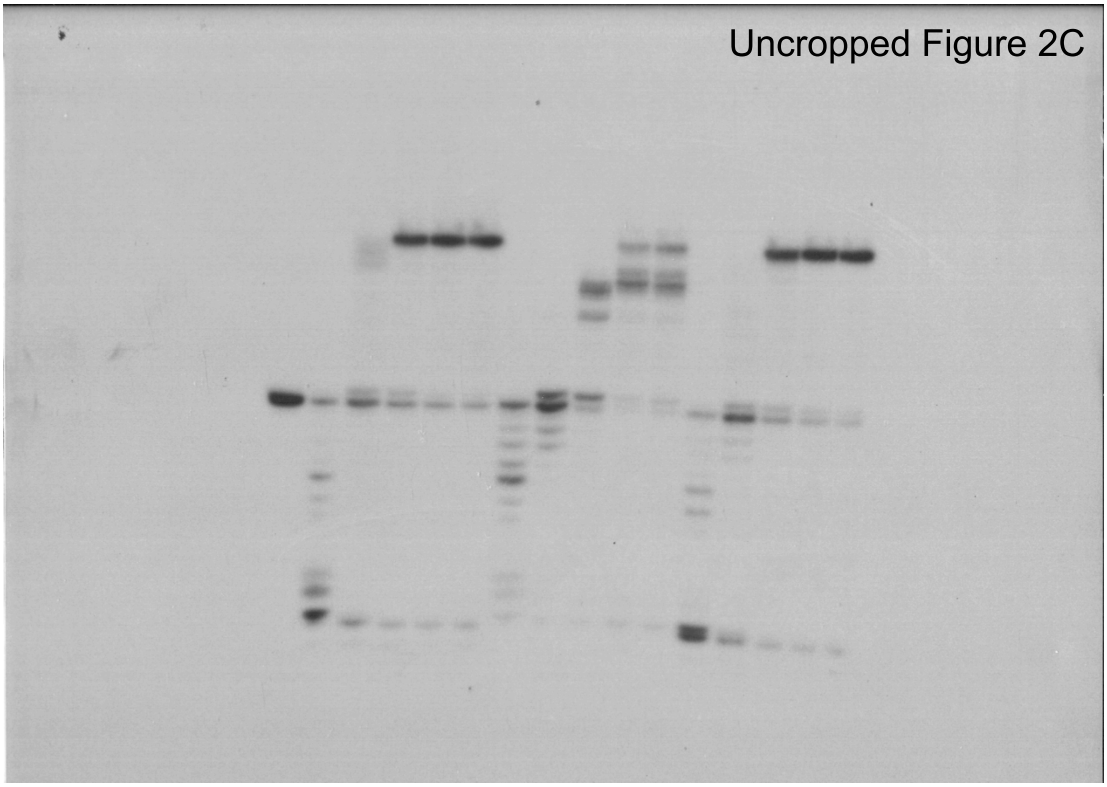

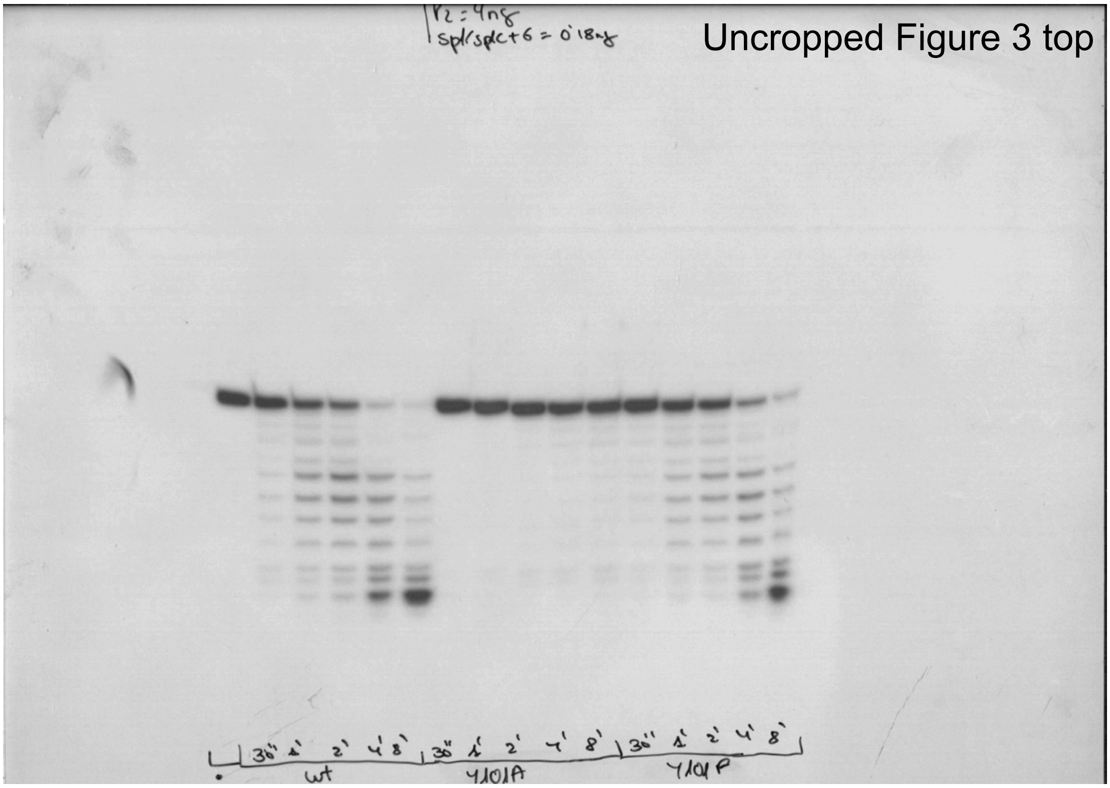

Supplementary Figure 8 (continued) : Uncropped gels for Figures 2 and 3

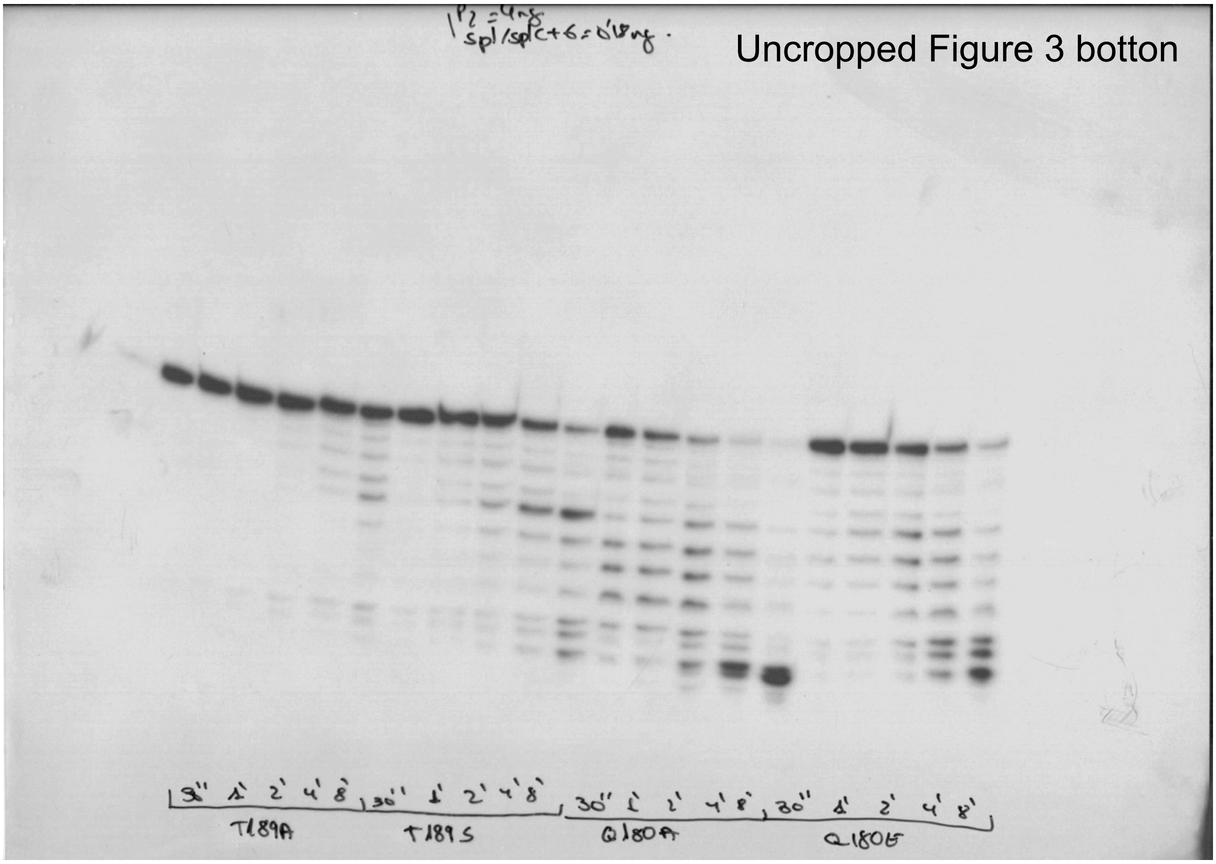

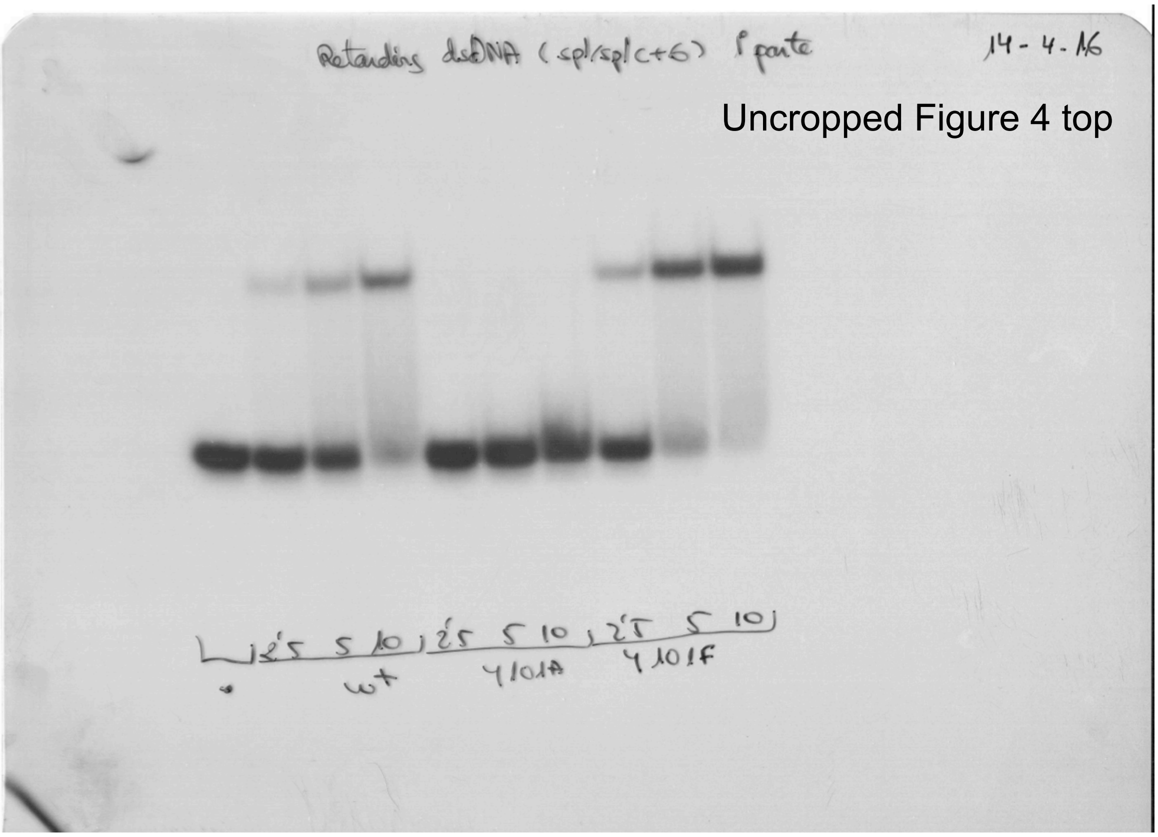

Supplementary Figure 8 (continued) : Uncropped gels for Figures 3 and 4

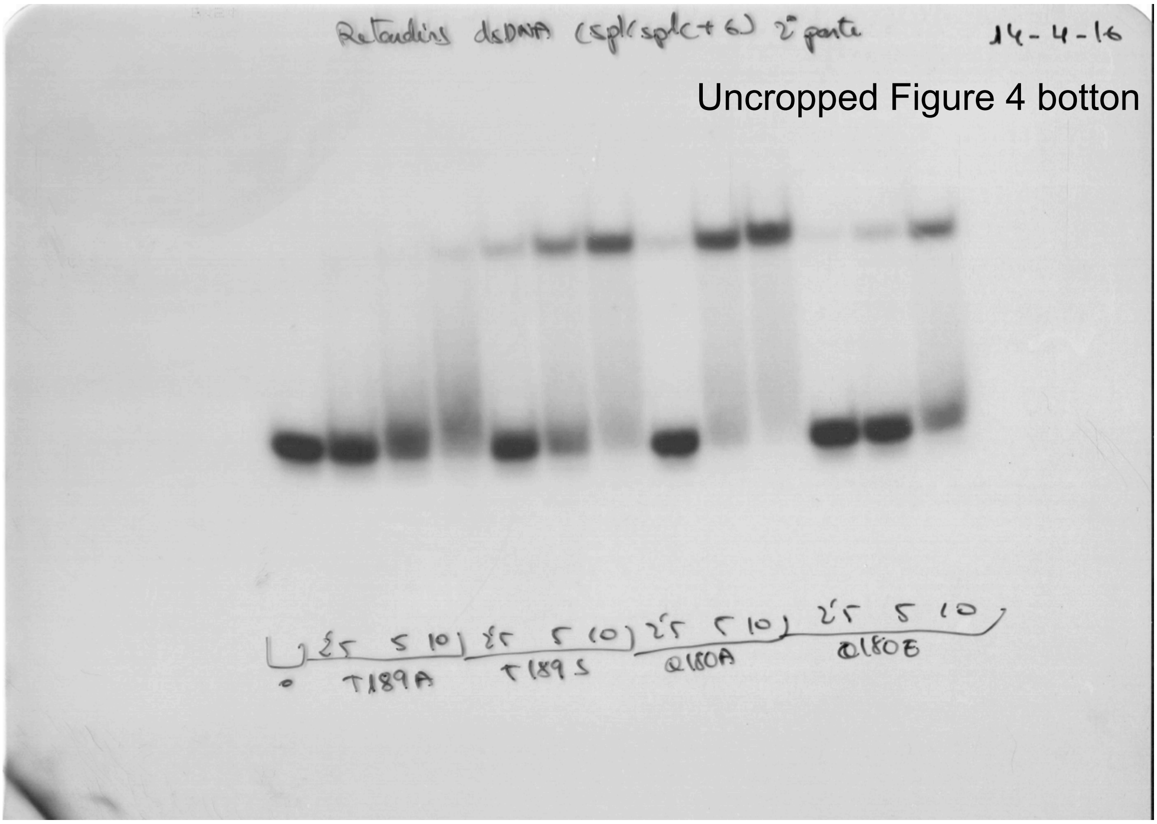

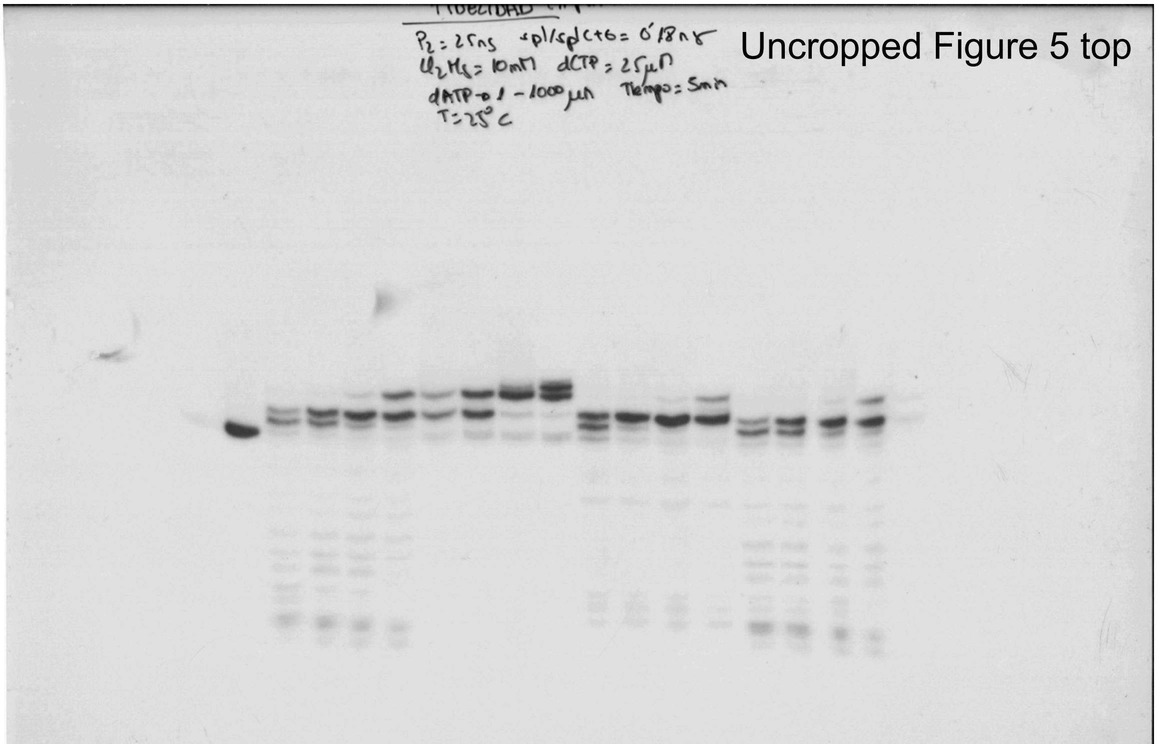

Supplementary Figure 8 (continued) : Uncropped gels for Figures 4 and 5

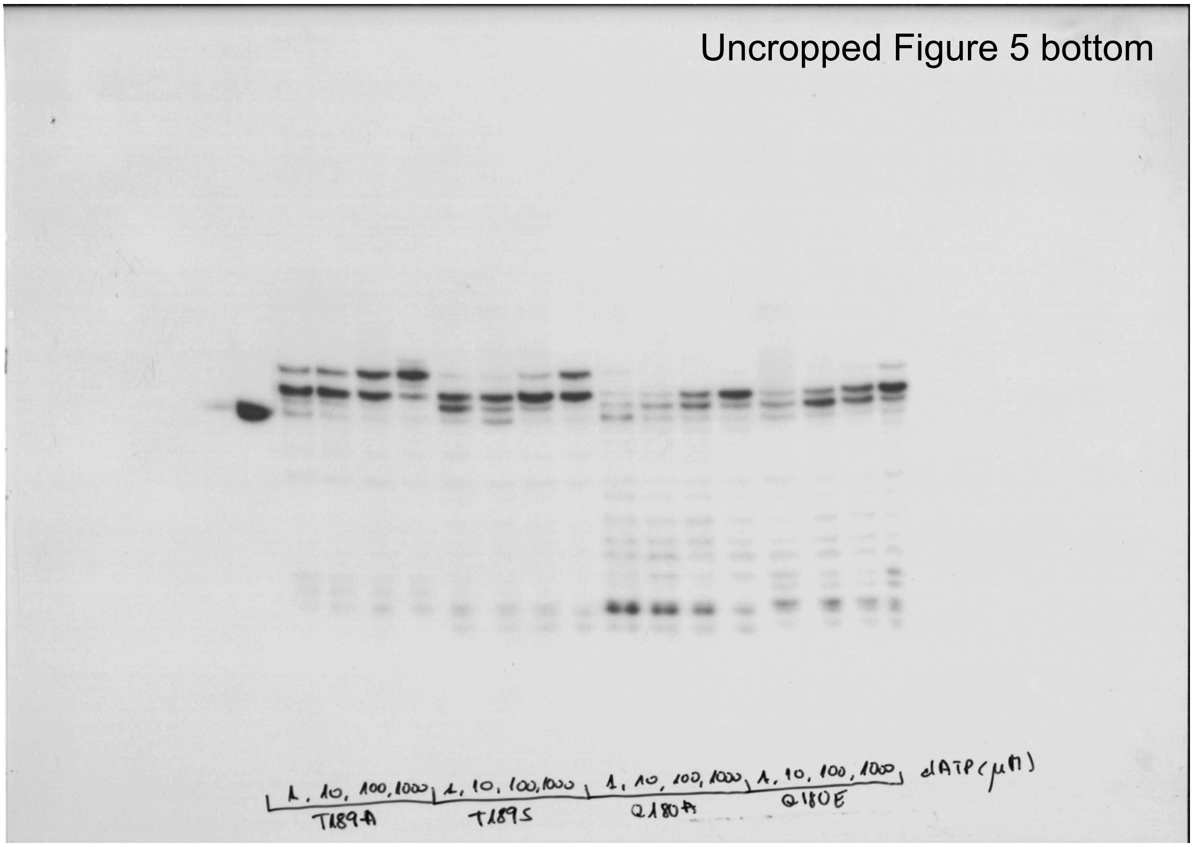

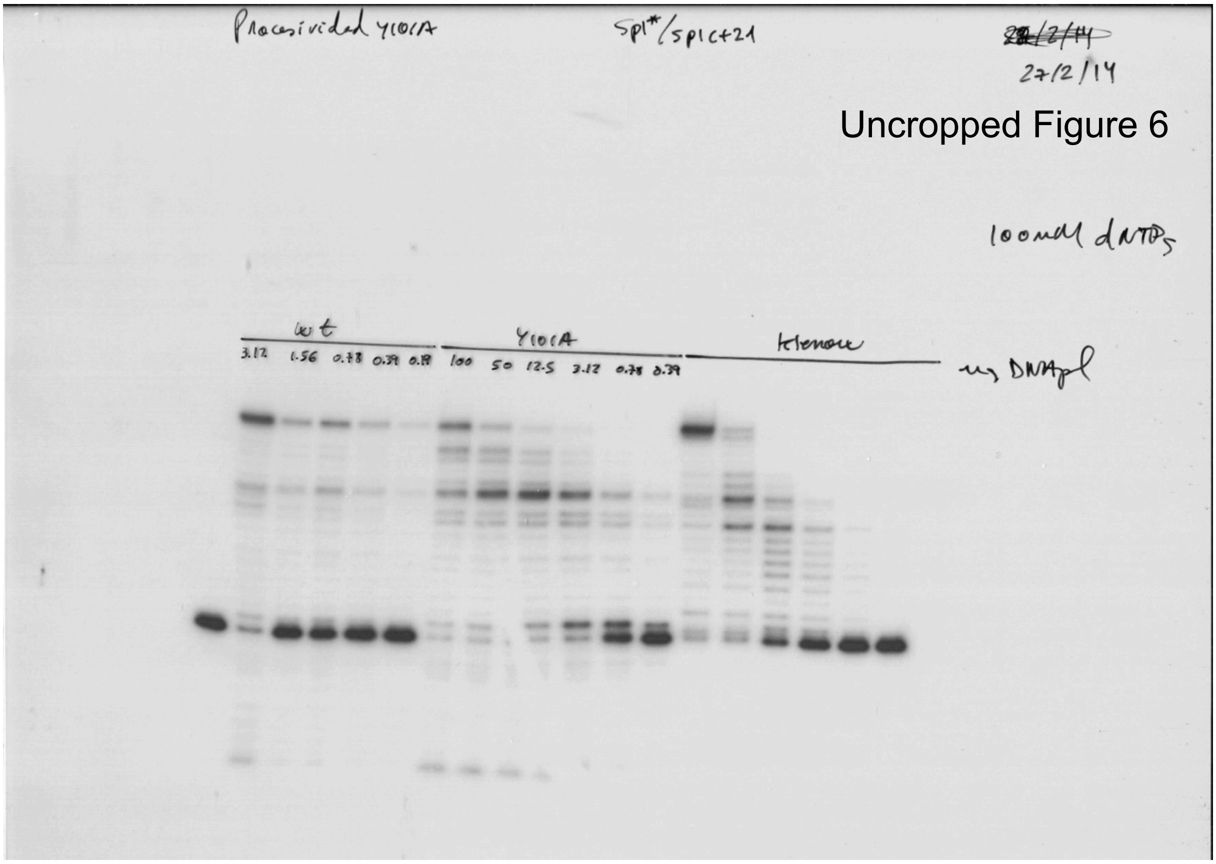

Supplementary Figure 8 (continued) : Uncropped gels for Figures 5 and 6

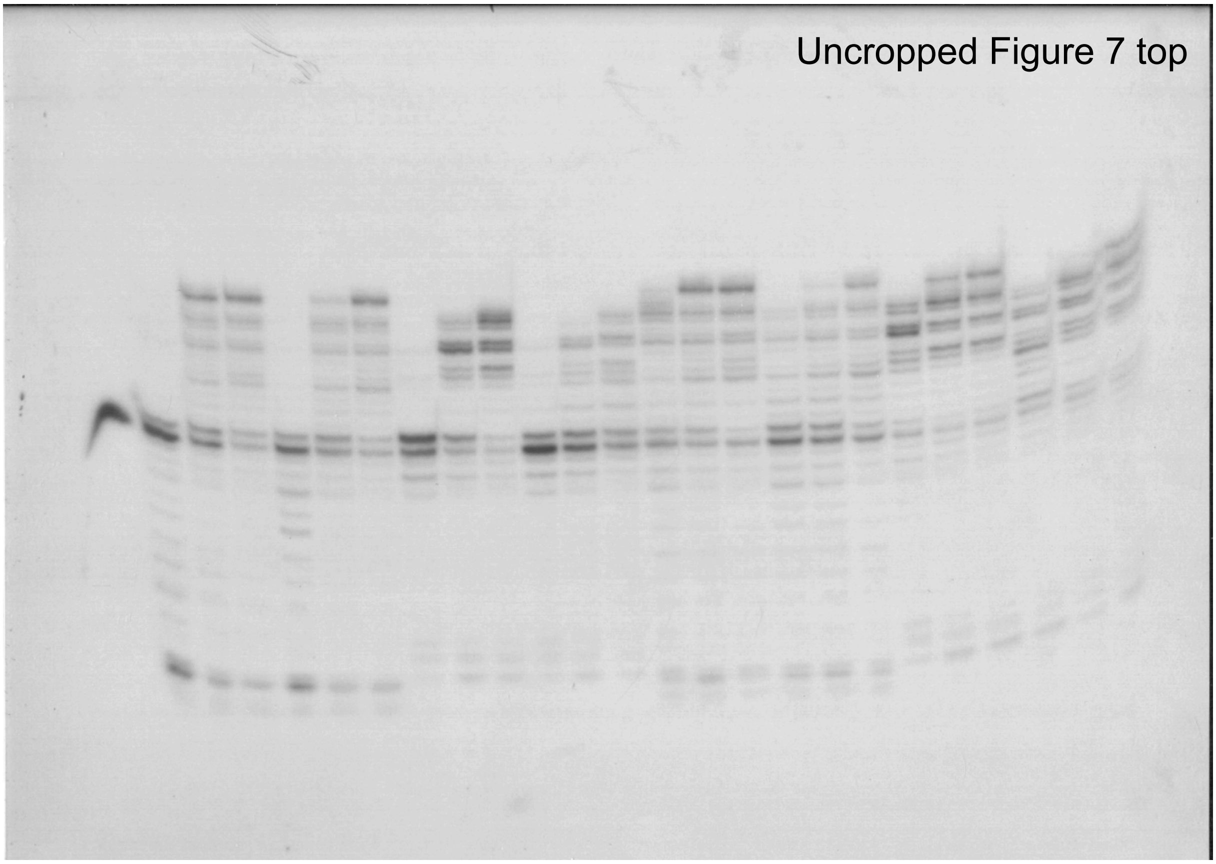

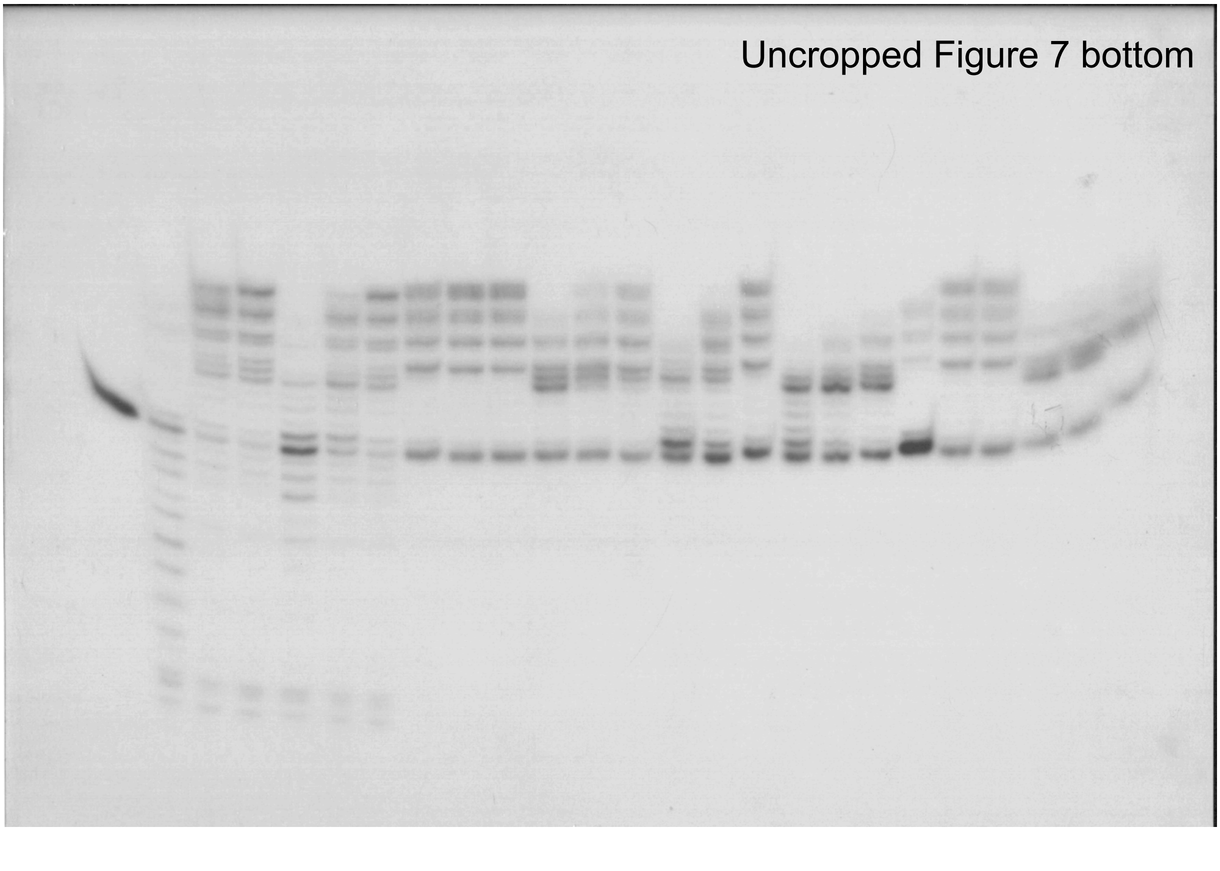

Supplementary Figure 8 (continued) : Uncropped gels for Figure 7

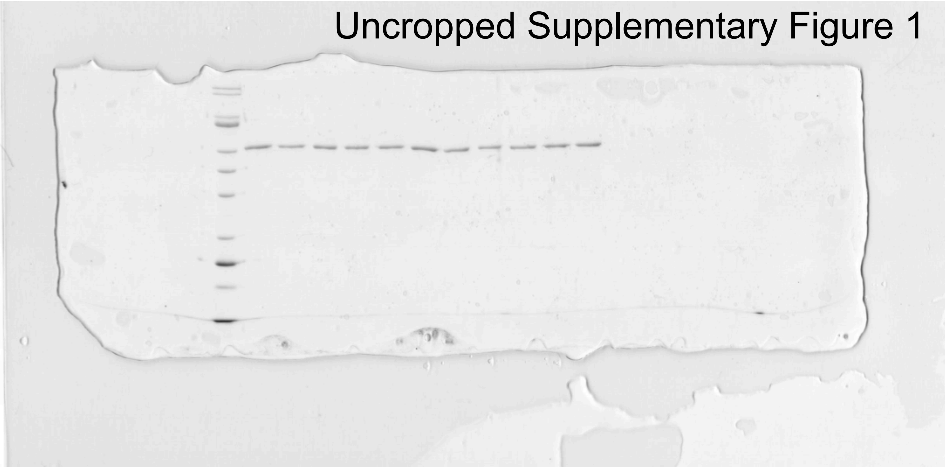

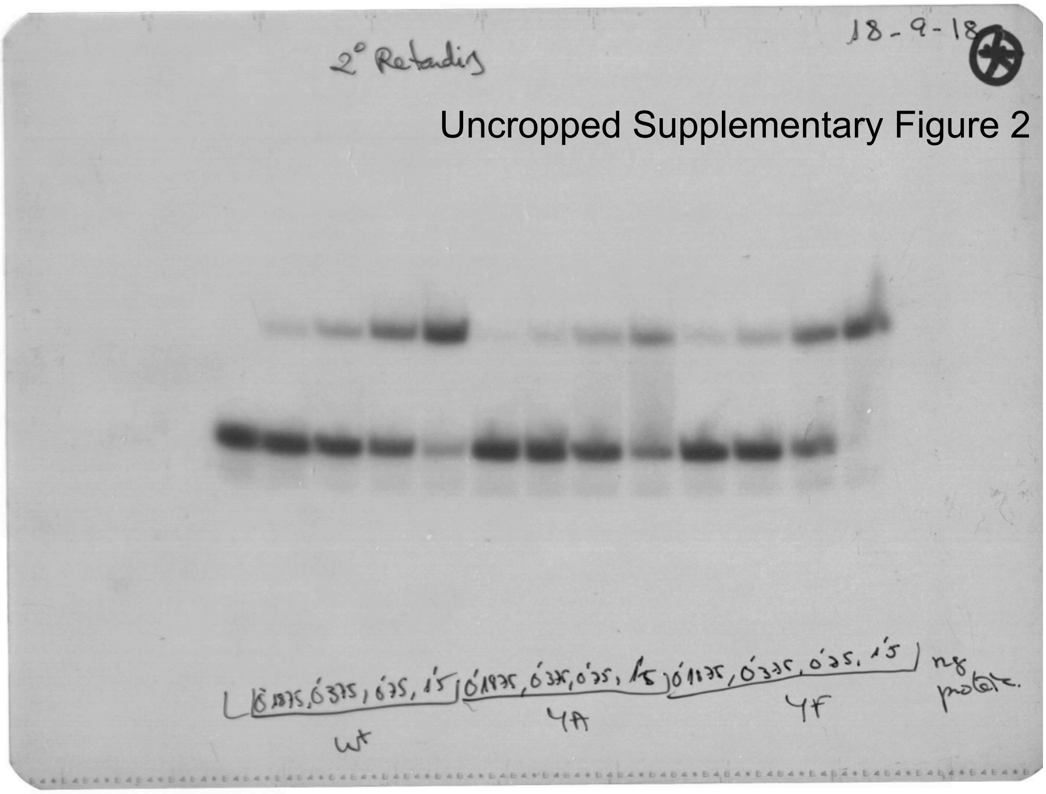

Supplementary Figure 8 (continued) : Uncropped gels for Supplementary Figures 1 and 2

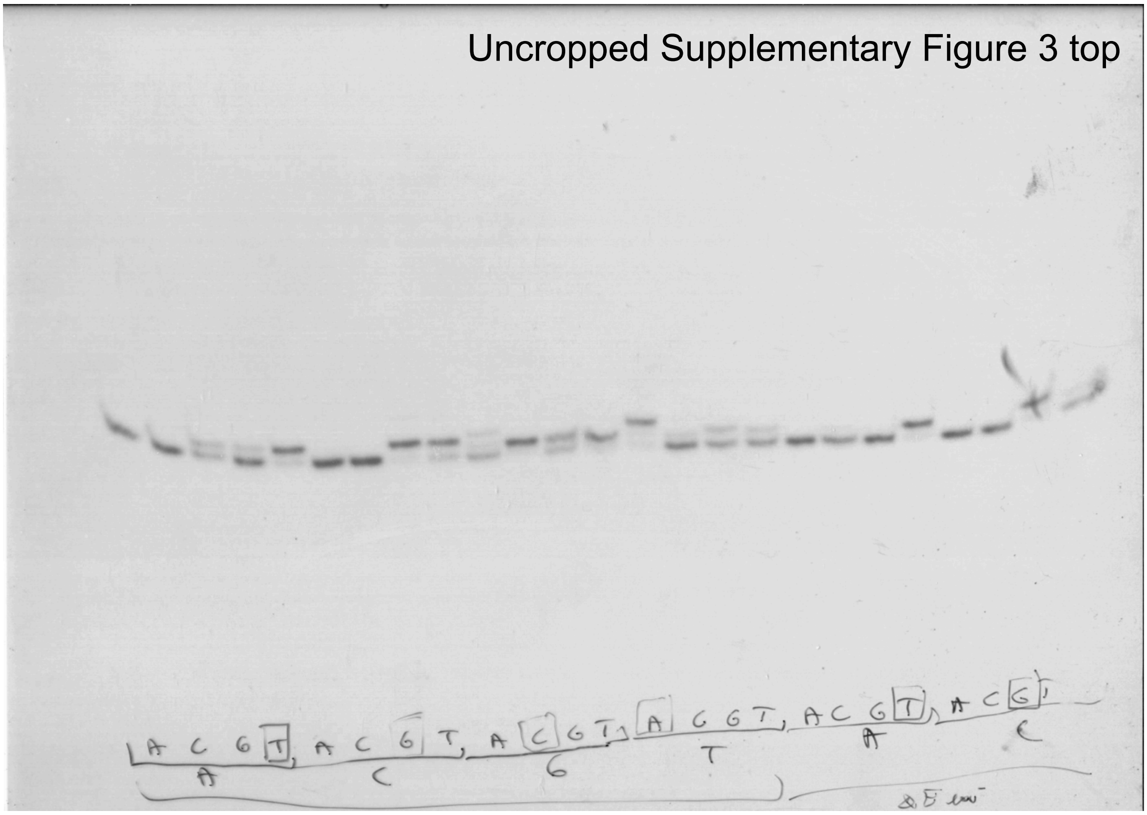

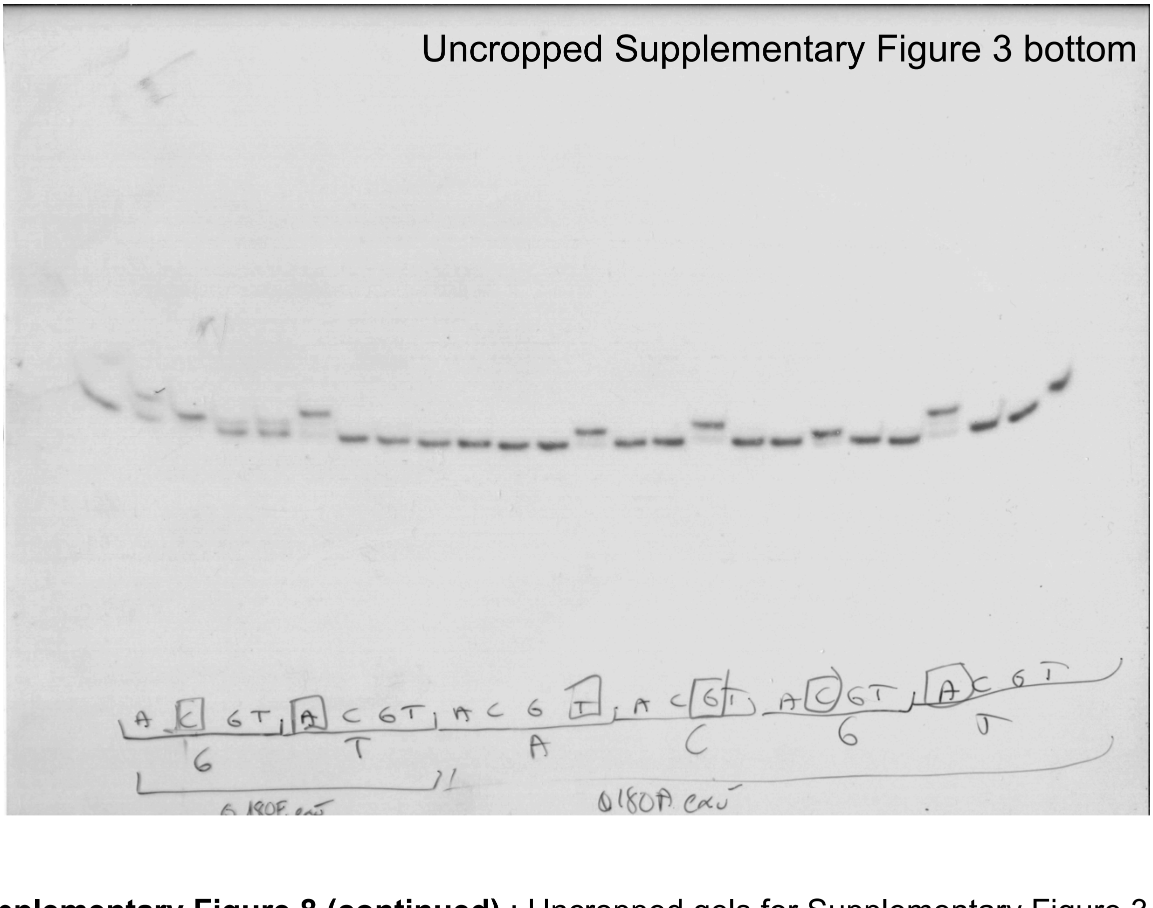

Supplementary Figure 8 (continued) : Uncropped gels for Supplementary Figure 3

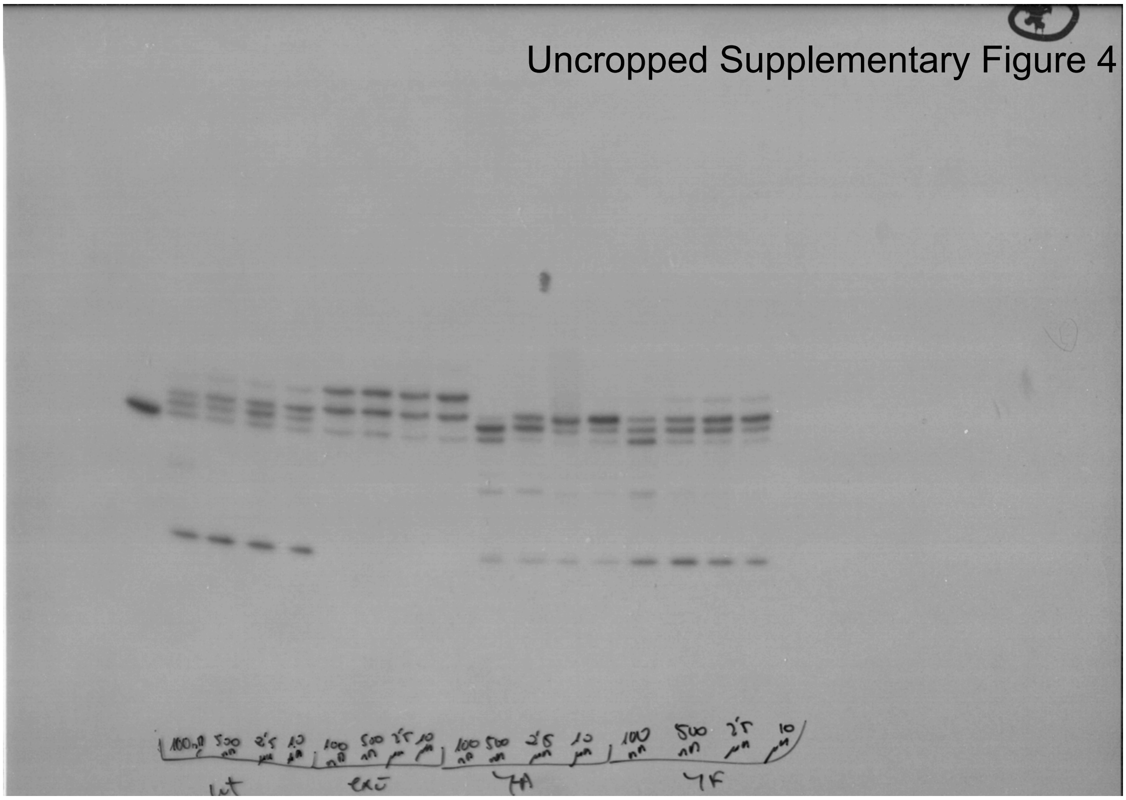

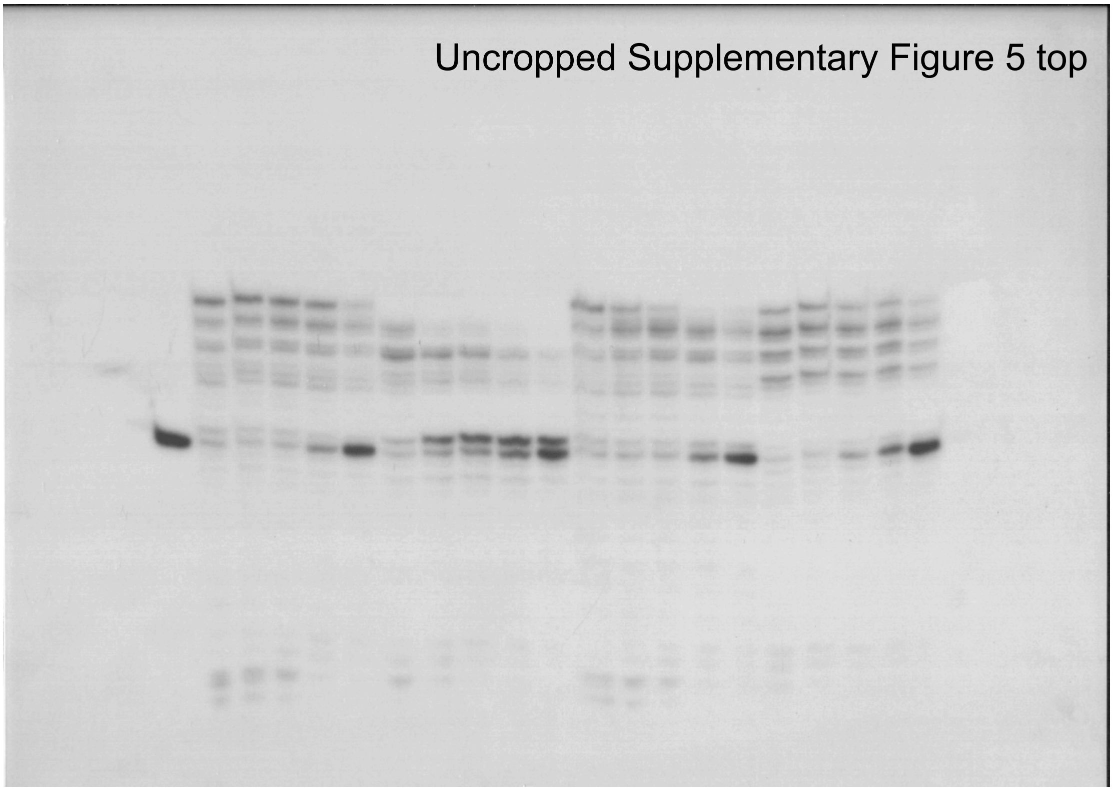

Supplementary Figure 8 (continued) : Uncropped gels for Supplementary Figures 4 and 5

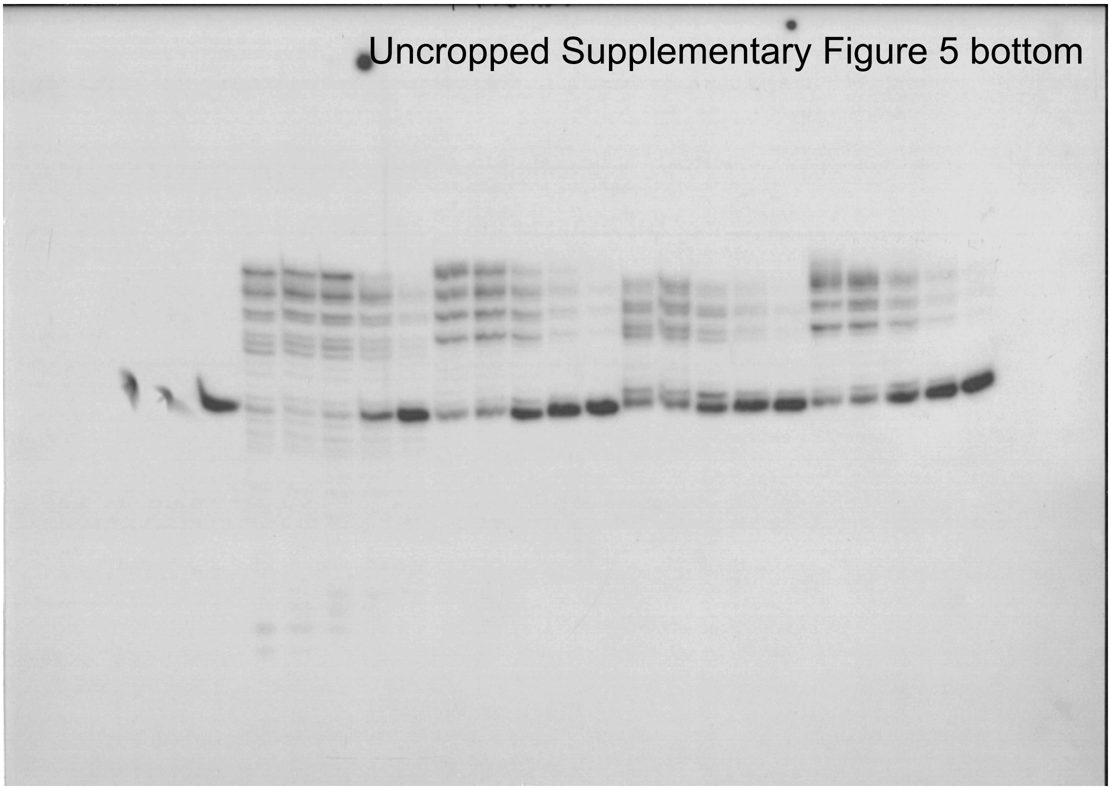

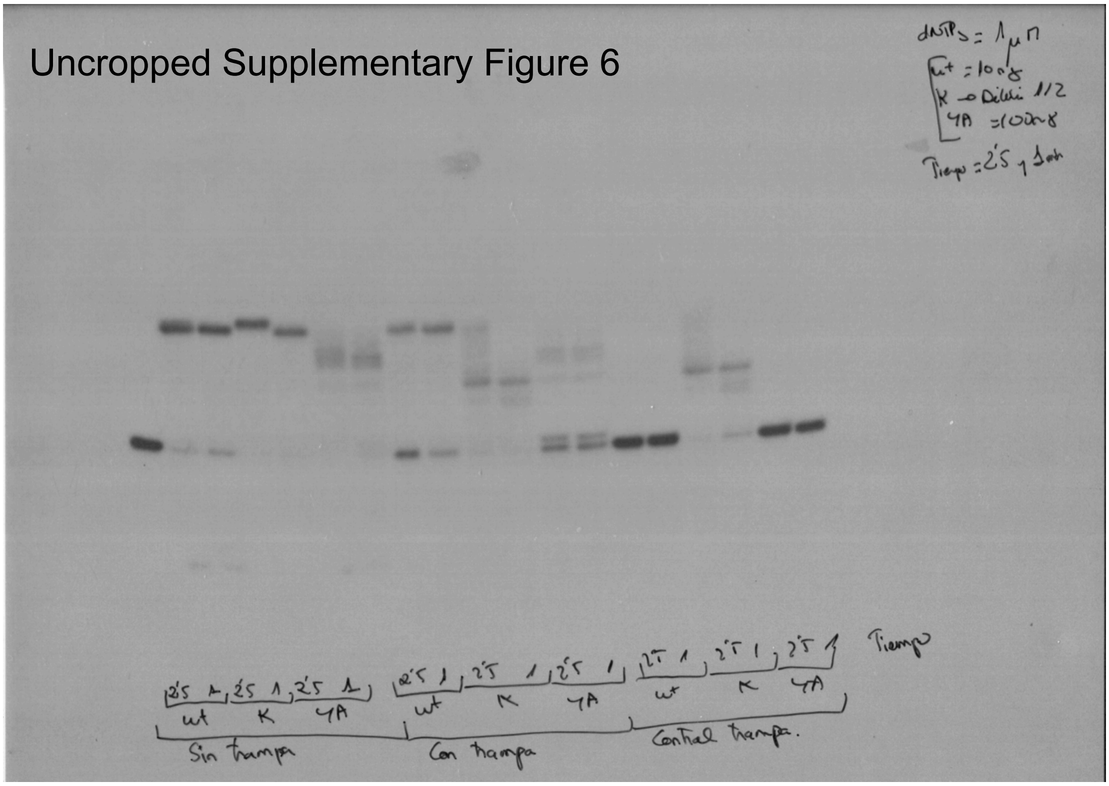

Supplementary Figure 8 (continued) : Uncropped gels for Supplementary Figures 5 and 6

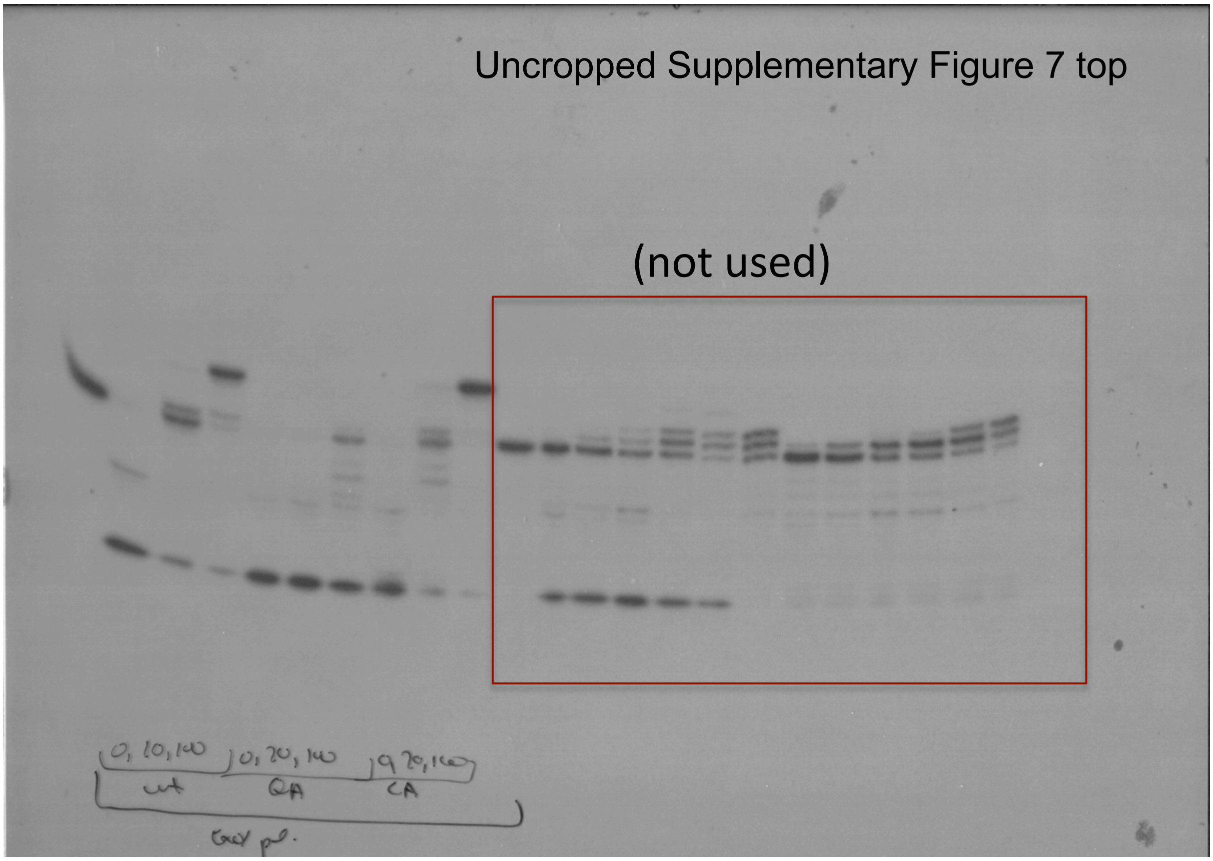

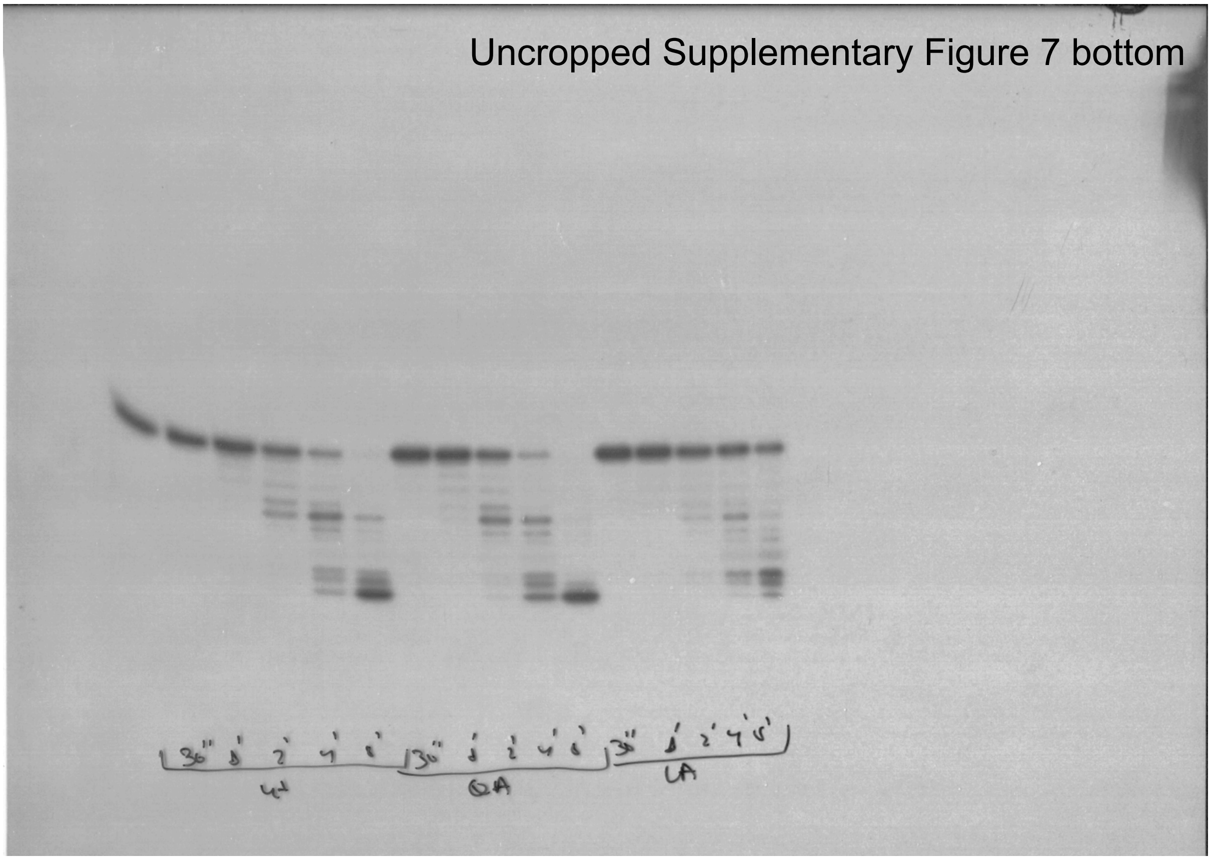

Supplementary Figure 8 (continued) : Uncropped gels for Supplementary Figure 7
